# Molecular forms of neurogranin in cerebrospinal fluid

**DOI:** 10.1111/jnc.15252

**Published:** 2020-12-17

**Authors:** Faisal Hayat Nazir, Elena Camporesi, Gunnar Brinkmalm, Tammaryn Lashley, Christina E. Toomey, Hlin Kvartsberg, Henrik Zetterberg, Kaj Blennow, Bruno Becker

**Affiliations:** ^1^ Institute of Neuroscience and Physiology Department of Psychiatry and Neurochemistry The Sahlgrenska Academy at the University of Gothenburg Mölndal Sweden; ^2^ Clinical Neurochemistry Laboratory Sahlgrenska University Hospital Mölndal Sweden; ^3^ Queen Square Brain Bank for Neurological Disorders Department of Clinical and Movement Neuroscience UCL Institute of Neurology London UK; ^4^ Department of Neurodegenerative Disease UCL Institute of Neurology Queen Square London UK; ^5^ UK Dementia Research Institute at UCL London UK

**Keywords:** CSF, heparin‐binding motif, neurogranin

## Abstract

Neurogranin (Ng) is a 78 amino acid neuronal protein and a biomarker candidate for Alzheimer's disease (AD). Ng has been suggested to bind to calmodulin and phosphatidic acid via its centrally located IQ domain. Ng is cleaved within this functionally important domain, yielding the majority of fragments identified in cerebrospinal fluid (CSF), suggesting that cleavage of Ng may be a mechanism to regulate its function. Up to now, Ng has been shown to be present in CSF as both C‐terminal fragments as well as full‐length protein. To obtain an overview of the different molecular forms of Ng present in CSF, we show by size exclusion chromatography (SEC), immunoblotting, immunoprecipitation, and MS that Ng is present in CSF as several molecular forms. Besides monomeric full‐length Ng, also higher molecular weight forms of Ng, and C‐terminal‐ and previously not identified N‐terminal fragments were observed. We found by immunodepletion that C‐terminal peptides contribute on average to ~50% of the total‐Ng ELISA signal in CSF samples. There were no differences in the overall C‐terminal fragment/total‐Ng ratios between samples from AD and control groups. In addition, we found that monomeric Ng and its C‐terminal fragments bind to heparin via a heparin‐binding motif, which might be of relevance for their export mechanism from neurons. Taken together, this study highlights the presence of several molecular forms of Ng in CSF, comprising monomeric full‐length Ng, and N‐ and C‐terminal truncations of Ng, as well as larger forms of still unknown composition.

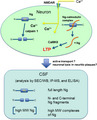

## Graphical abstract

C‐terminal fragments, along with full‐length forms of neurogranin (Ng) have been previously found at higher concentration in CSF from Alzheimer´s disease patients. Calpain 1 cleaves Ng in its IQ domain upon activation by Ca ions. This IQ domain is essential for modulating calmodulin and CaMKII signaling toward LTP. Using SEC, western blotting, and IP‐MS, we show that N‐terminal Ng and high molecular weight forms of Ng can be also be found in CSF. These Ng forms may have physiologic relevance and be of importance for the understanding of pathologic processes in brain disorders and for the development of specific biomarker assays.

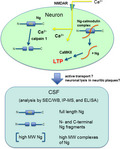

**Abbreviations**
aa: amino acid
BSA: bovine serum albumin
CaM: calmodulin
CNS: central nervous system
CSF: cerebrospinal fluid
DMP: dimethyl pimelimidate dihydrochloride
ELISA: enzyme‐linked immunosorbent assay
FA: formic acid
GAP43: growth‐associated protein 43
HRP: horseradish peroxidase
I: isoleucine
IP: immunoprecipitation
LC‐MS/MS: liquid chromatography‐tandem mass spectrometry
LTP: long‐term potentiation
m/z: mass‐to‐charge ratio
mab: monoclonal antibody
MALDI: matrix‐assisted laser desorption/ionization
MS: mass spectrometry
MW: molecular weight
NFL: neurofilament light protein
Ng: neurogranin
PA: phosphatidic acid
PBS‐T,PBS with 0.05%: Tween20
PREP: prolyl‐endopeptidase
Q: glutamine
RRID: resource identifiers research
SD: standard deviation
SDS‐PAGE: sodium dodecyl sulfate–polyacrylamide gel electrophoresis
SEC: size exclusion chromatography
TBS: Tris‐buffered saline

## 1 INTRODUCTION

Neurogranin (Ng) is a 78 amino acid (aa) post‐synaptic protein known to be expressed in cerebral cortex, amygdala, caudate, putamen, and the hippocampus in the brain (Alvarez‐Castelao & Schuman, 2015; Represa et al., 1990). Outside the central nervous system (CNS), Ng is highly expressed in platelets, moderately in B‐lymphocytes, and low expression is detected in lung, spleen, and bone marrows (Diez‐Guerra, 2010; Glynne et al., 2000; Gnatenko et al., 2003). Ng has been suggested to bind intracellularly to calmodulin (CaM) (Prichard et al., 1999) and phosphatidic acid (PA) (Domínguez‐González et al., 2007). Ng binds to CaM via its IQ motif (aa 33–46), so‐called because of the presence of the aa isoleucine (I) and glutamine (Q) at positions 33 and 34 of the aa sequence of Ng; this IQ motif is well conserved among other CaM‐binding proteins, including growth‐associated protein 43 (GAP‐43) and Purkinje cell protein 4 (Pcp4, also called PEP‐19) (Bahler & Rhoads, 2002; Diez‐Guerra, 2010).

Ng plays an important role in long‐term potentiation (LTP) by modulating CaM signal transduction in response to intracellular calcium concentrations to enhance synaptic plasticity. LTP is suggested to be essential for the formation of long‐term memories (Diez‐Guerra, 2010; Gerendasy et al., 1995; Huang et al., 2004). It has been observed that Ng expression is reduced in the hippocampus of mice with age, which has been implicated in age‐related CNS dysfunction (Mons et al., 2001). Furthermore, Ng knockout mice have deficits in learning and memory (Pak et al., 2000), whereas Ng up‐regulation restores LTP and cognitive performance (Zhong et al., 2009; Alzoubi et al., 2005).

**FIGURE 1:**
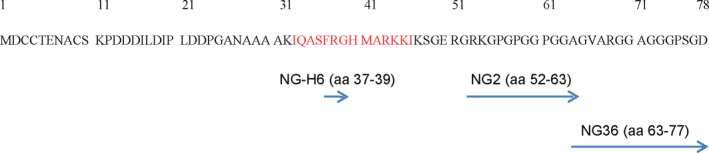
Ng amino acid (aa) sequence. The aa marked in red text indicates the IQ domain in the Ng sequence. The experimentally estimated epitopes for NG‐H6 and for the NG2 monoclonal antibody and the immunogen for the NG36 monoclonal antibody are indicated (blue arrows)

Research from our group has shown that Ng can be detected at increased concentrations in cerebrospinal fluid (CSF) from Alzheimer's disease (AD) patients, both as full‐length protein (Thorsell et al., 2010) and as C‐terminal fragments (Kvartsberg et al., 2015). Elevated Ng concentrations can also be found in CSF of Creutzfeldt–Jacob disease patients (Blennow et al., 2019; Villar‐Pique et al., 2020). Molecular characterization of Ng in CSF suggested that Ng is present as a ~12 kDa protein as detected by immunoblotting (Thorsell et al., 2010) of immunoprecipitated CSF. However, mass spectrometry (MS)‐based characterization of CSF Ng has revealed the existence of several C‐terminal endogenous fragments of the protein. In total, 15 Ng peptides that spanned mainly the C‐terminal half of the molecule were identified in CSF (Kvartsberg, Duits, et al., 2015). Most C‐terminal fragments have their N‐terminal ending near or in the IQ domain (aa 33–46) and their C‐terminal endings at or near the C‐terminal end of the full‐length Ng (aa 78) (see Figure 1). It has been shown that these fragments were not the products of in vitro degradation of Ng in CSF (Kvartsberg et al., 2015). Therefore, we investigated potential Ng‐cleaving enzymes and found that calpain‐1 and prolyl‐endopeptidase (PREP) cleaved Ng and suggested that those cleavages were generating those endogenous Ng fragments (Becker et al., 2018). Peptides covering the N‐terminal half of Ng have thus far not been detected in CSF; the search for such peptides has been hampered by the lack of potent and specific antibodies that could be used for immunoprecipitation.

A study on bovine brain extracts using fast protein liquid chromatography (FPLC) followed by immunoblotting suggested that most of the Ng eluted as dimers and higher oligomers (Baudier et al., 1991). Furthermore, MS data on TBS brain extracts showed a small peak at m/z 15,318, which may correspond to a Ng dimer. In CSF, using mass spectrometry of immunoprecipitated samples, only minute amounts of full‐length Ng have been suggested to be present (Kvartsberg, Duits, et al., 2015).

A recent study from our group indicated that Ng processing is increased in AD brain tissue and therefore, the ratio of peptide‐to‐total full‐length Ng also increased for several endogenous Ng peptides (Kvartsberg et al., 2019). The molecular forms of Ng and its processing in brain tissue may be reflected in CSF, which thus may allow monitoring disease progression. Proteolytic processing may regulate Ng function by cleaving its IQ domain and may also change higher order structures of Ng (oligomers, or complexes of Ng with other proteins).

As a first step to understand more about those processes, we set out to identify which molecular forms of Ng exist in CSF. First, we used size‐exclusion chromatography (SEC) of CSF pools to separate the molecular forms of Ng in CSF. For the first time, using a modified immunoblotting method which better retained small fragments of Ng on nitrocellulose membranes, we were able to show the presence of several forms of Ng in CSF, namely higher molecular weight forms, full‐length Ng and C‐terminal fragments. Using immunoprecipitation and MS, we subsequently show that these fragments comprised both N‐ and C‐terminal Ng fragments, most of them generated by cleavage between aa 42 and aa 43, which is a reported calpain‐1 cleavage site (Becker et al., 2018). Second, in an effort to estimate the proportion of C‐terminal Ng fragments to total‐Ng content, we depleted full‐length Ng and mainly N‐terminal Ng fragments from several individual AD and control CSF samples and measured by sandwich ELISA the concentration of the remaining Ng which represented mainly the C‐terminal Ng fragments, allowing estimation of the proportion of C‐terminal fragments to total‐Ng content. On average, this proportion amounted to about 50% and it was the same for the control as well as the AD sample set. This may give an explanation why Ng ELISAs targeting either full‐length Ng or the sum of C‐terminal and full‐length Ng have been reported to have the same predictive power for distinguishing AD from controls (Willemse et al., 2018). We finally show that full‐length Ng and the C‐terminal fragments contained a functional heparin‐binding motif, which, similar as for tau, may allow their export from neurons via the unconventional transport mechanism (Katsinelos et al., 2018).

## 2 MATERIALS AND METHODS

### 2.1 Ethical permit

Pooled CSF samples and CSF collected from individuals used in this study were de‐identified clinical samples from the Neurochemistry Laboratory at the Sahlgrenska University Hospital, Mölndal, Sweden, following a procedure approved by the Ethical Committee at the University of Gothenburg (EPN, 11. Aug. 2014). Human post‐mortem brain tissue was obtained through the brain donation program at Queen Square Brain Bank for Neurological Disorders, Department of Clinical and Movement Neurosciences, Institute of Neurology, University College London (UCL). Human brain tissues were used in accordance with the Helsinki declaration and were approved by the regional ethics committees at UCL and the University of Gothenburg. This study was not pre‐registered.

### 2.2 CSF collection and ELISA assays for amyloid beta 1–42, t‐tau and p‐tau

CSF samples were collected by lumbar puncture before noon following standard operating procedures. CSF samples were collected at ambient temperatures into polypropylene tubes (Cat# 62.9924.284, Sarstedt, Germany) using 22G spindle needle. Samples were centrifuged at 1,750 *g* for 5 min at 21°C and aliquoted to 2 mL tubes (Cat# 021‐4204‐500, Elkay Laboratory Products, UK) and stored at −80°C within 1–4 hr of collection. The samples were stored for 3 to 6 years before utilized in experiments.

In experiments involving SEC, we have used pools of CSF (*N* = 4) to obtain sufficient sample volumes. These were obtained by pooling anonymized clinical samples of CSF (healthy individuals, as well as patients suffering from various neurodegenerative and neurological diseases). In the immunodepletion experiment, we used CSF samples collected from 20 individuals. The samples were assigned with help of CSF biomarkers for AD (Table 1) to a control group (Ctrl 1–9) and AD group (AD 1–11), based on low Aβ42 =<650 and elevated total‐tau (t‐tau) >=375 for AD‐like samples. For Ctrl 1, which had an Aβ42 below the cut‐off for normal values, we took as additional selection criterion phosphorylated‐tau (p‐tau) 181 (AD: p‐tau > 52 and >80 for ages over 60).

**TABLE 1:** Demographic and biochemical data on individual CSF samples used to determine the proportion of C‐terminal Ng fragments to total‐Ng

CSF sample	Aβ42 (ng/L)	t‐tau (ng/L)	p‐tau181 (ng/L)	Ng (ng/L)	Age (yrs)
Ctrl 1	585	170	25	72	88
Ctrl 2	769	126	28	140	35
Ctrl 3	831	337	54	137	54
Ctrl 4	693	250	32	100	68
Ctrl 5	716	322	35	60	68
Ctrl 6	777	298	41	226	58
Ctrl 7	693	236	35	180	61
Ctrl 8	651	127	19	74	61
Ctrl 9	682	259	35	231	71
AD1	544	519	73	177	78
AD2	533	588	71	235	76
AD3	559	667	70	200	55
AD4	549	738	71	175	81
AD5	275	678	77	203	75
AD6	483	665	70	175	69
AD7	393	599	67	131	80
AD8	509	806	76	179	83
AD9	410	698	76	193	75
AD10	536	656	74	245	77
AD11	461	685	67	179	85

Ctrl, control CSF; AD, AD‐like CSF.

CSF Aβ42, t‐tau, and p‐tau were analyzed with INNOTEST ELISA (Fujirebio Europe, Ghent, Belgium). This experiment was blinded for the experimenter since the samples were de‐identified.

### 2.3 Ultrafiltration

For the concentration of CSF prior to SEC, we used four pools of CSF with Ng concentrations between 849–1271 pg/mL. They were used for ELISA (Figure 3), non‐reducing western blots after SEC (Figure 4 and Figure S2) , IP‐MS (Figure 5a), and SEC‐reducing western blot (Figure S3). Pooled CSF was placed in Amicon® Ultracentrifugal tubes (YM‐3 membrane, Cat# UFC900308, Merck) with a molecular weight cut‐off (MWCO) of 3,000 Da. The centrifugations were performed at 3,000 *g* for 1 hr at 4°C in a swinging bucket rotor to obtain a retentate volume of about 10% of the initial CSF volume. The about 10‐fold concentrated CSF samples were aliquoted and thereafter stored at −80°C until further analysis.

### 2.4 Size exclusion chromatography of CSF concentrates

Size exclusion chromatography (SEC) was performed to separate molecular species of Ng in CSF using 50 mM Tris, pH 7.5, 10% (w/w) glycerol as elution buffer. A 300 × 10 mm Superdex 200 column (GE Healthcare) was calibrated using a SEC size marker kit (Cat# MWGF70‐1KT, Merck) containing blue dextran (marking void volume; 2,000 kDa), bovine serum albumin (66 kDa), carbonic anhydrase (from bovine erythrocytes; 29 kDa), cytochrome C (from horse heart; 12.4 kDa), and aprotinin (from bovine lung; 6.5 kDa). An aliquot of 1 mL of pooled concentrated CSF was then injected onto the Superdex 200 column in cold conditions (4°C). Fractions of 0.5 mL were collected at a flow rate of 0.4 mL/min. The collected fractions were processed for western blotting and left‐overs of the fractions were stored at −80°C.

### 2.5 Antibodies, peptides, recombinant Ng protein, pooled CSF, and brain extract

C‐terminal region‐specific monoclonal Ng antibodies, NG36 and NG2 were prepared in‐house by immunizing Balb/c mice (8 week old) with the KLH‐conjugated peptides corresponding to human Ng sequence aa 63–77 and aa 52–63 (Caslo, Denmark) (Becker et al., 2018; Kvartsberg et al., 2019). The exact position of the epitope for NG36 has not been determined experimentally, but in a previous paper (Kvartsberg et al., 2019), we showed that it captures the same C‐terminal Ng peptides as NG2 in human brain tissue, including peptides ending at position 75, meaning that the epitope is likely in the region of Ng63‐75. A monoclonal Ng antibody, NG‐H6, with reported immunogen Ng1‐50 and previously mapped epitope aa 37–39 (Höglund et al., 2020), was purchased from Santa Cruz Biotechnologies (Cat# sc‐514922). A polyclonal Ng antibody Ab270 was a gift from Prof. Kuo‐Ping Huang. Mouse anti‐rabbit IgG‐horseradish peroxidase (HRP) and mouse IgGκ‐binding protein‐HRP were purchased from Santa Cruz Biotechnologies (Cat# sc‐2357, RRID:AB_628497 and Cat# sc‐516102, RRID:AB_2687626, respectively). GAP‐43 monoclonal and polyclonal antibodies were purchased from Abcam (Cat# ab75810, RRID:AB_1310252) and Nordic BioSite (Cat# ABB‐135), respectively. Neurogranin peptides were synthesized by Caslo (Denmark), and full‐length recombinant Ng (aa 1‐78) was prepared as described previously (Becker et al., 2018). The pooled CSF used in the ultrafiltration and western blot experiments had concentrations of 1,271 pg/mL Ng, 423 pg/mL Aβ42, 298 pg/mL total tau, and 43 pg/mL of p‐tau, as determined by ELISA. Pooled samples of TBS extracts from AD and control human brains were used as standards of human brain Ng in western blots. Briefly, samples of temporal cortex of control and AD brains (100 mg ± 20 mg) were homogenized in 1 mL TBS buffer (20 mM Tris‐HCl, 137 mM NaCl, pH  7.6) and protease inhibitor (Complete Protease inhibitor cocktail, Cat# 11697498001; Roche Diagnostic GmbH) using the Tissue Lyser II (Qiagen). After homogenization, samples were centrifuged for 1 hr at 31,000 *g* at + 4°C. The supernatant (soluble fraction) was aliquoted and stored frozen at −80°C.

### 2.6 Gel electrophoresis and western blot

The samples were prepared by adding XT sample buffer containing lithium dodecyl sulfate (LDS) with or without reducing agent (all buffers from Bio‐Rad Laboratories) followed by heating to 70°C for 10 min. The samples were loaded onto 4%–12% Bis‐Tris gels, and run using MES buffer (Cat# 1610789; Bio‐Rad laboratories); as molecular weight markers, SeeBlue Plus2^™^ (Cat# LC5925; ThermoFisher Scientific) pre‐stained protein standard was used. Thereafter, the gels were equilibrated with 1 × transfer buffer (NuPAGE™; Cat# NP0006‐1; ThermoFisher Scientific) with added methanol (20% v/v). The separated proteins were blotted onto a nitrocellulose membrane (Protran 0.2 μm; Cat#10600001; Amersham) using a semi‐dry blot apparatus. During blotting, the current was kept constant at 90 mA (approx. 0.7 mA/cm^2^) for 1 hr; thereafter the blots were air‐dried overnight followed by fixing of the blotted proteins using 0.4% formaldehyde (Cat#28906; ThermoFisher Scientific) in PBS for 30 min at 20‐25°C. After three washes with PBS (5 min each), the membranes were blocked with 5% non‐fat dry milk (Cat# 170–6404; Bio‐Rad Laboratories) in PBS‐T (PBS with 0.05% Tween 20) followed by incubation with anti‐Ng antibodies in 0.1% BSA in PBS‐T. After washing, membranes were incubated with HRP‐conjugated anti‐mouse IgG antibody (Cat# 7076, RRID:AB_330924; Cell Signalling Technologies), or with mouse IgGκ‐binding protein‐HRP conjugate (Cat# sc‐516102, RRID:AB_2687626; Santa Cruz Biotechnology). For protein detection, ECL Select (Cat# RPN2235; GE Healthcare) was used according to standard procedures. Images were acquired using Fujifilm LAS‐3000 imager system (Fujifilm Medical Systems). The experiments were not blinded for the experimenter.

### 2.7 Immunoprecipitation (IP)

Ng and Ng peptides were immunoprecipitated from a concentrated CSF pool and after SEC fractionation of that pool. Briefly, NG36 (in‐house) and NG‐H6 (Cat# sc‐514922, Santa Cruz Biotechnology) monoclonal antibodies were used as capture antibodies (epitopes located in the C‐ and N‐terminal half of Ng, respectively). For each sample to be analyzed (2.5 mL), three µg of each antibody (or the same volume of PBS for control beads) were added separately to aliquots of 50 µL of magnetic Dynabeads M‐280 (Cat. #11202D, RRID:AB_2783009; ThermoFisher Scientific) and incubated 75 min on a rocking platform at 20‐25°C. The beads were washed three times with triple volumes of PBS and thereafter combined (however, control beads were kept separate). The beads were then exposed to 150 µL of 20 mM dimethyl pimelimidate dihydrochloride (DMP, Cat# 21666; ThermoFisher Scientific) cross‐linker in 0.2 M triethanolamine (pH 8.2; Cat# 90279; Merck). After 30 min incubation at 20‐25°C, the reaction was quenched by the addition of 7.5 µL 1M Tris‐HCl (Trizma Base, pH 7.5, Cat# T2319; Merck). The cross‐linked beads were subsequently washed in PBS containing 0.05% Triton‐X 100 and blocked with 10‐fold diluted Roti‐Block (Cat# A151.1; Carl Roth) for 90 min, with shaking at 20‐25°C. After washing with PBS, the conjugated beads were resuspended in 100 µL PBS. For the IP‐binding reactions, 250 µL of the concentrate CSF pool was used and diluted with PBS to 2.5 mL; for IP of SEC fractions, 250 µL of each of three peak fractions (by western blot) was combined and adjusted with PBS to the final same volume of 2.5 mL. IP of the CSF samples (2.5 mL) diluted as described was performed by adding 100 µL of the antibody‐conjugated bead suspension, 0.05% Triton‐X 100 and Complete Protease inhibitor Cocktail (Cat # 4693116001; Merck) and incubating overnight on a rocking platform at +4°C. The day after, the samples with the magnetic beads were transferred to a KingFisher™ Flex System (ThermoFisher Scientific). The beads were washed twice with 1 mL 0.05% Triton‐X 100 in PBS, twice with each 1 mL PBS and once with 0.5 mL 50 mM ammonium hydrogen carbonate. Each washing step was for 2 min with shaking. Ng and Ng fragments were eluted from the beads by adding 100 µL 0.5% formic acid (FA) for 10 min, with shaking. The eluates were transferred to 0.5 ml Costar microcentrifuge tubes (Cat# 3207; Corning®), dried in a vacuum centrifuge, and stored at −20°C pending analysis by MS. In one type of experiment, only C‐terminal NG36 antibody was used for IP.

### 2.8 MS analysis of immunoprecipitates

The dried immunoprecipitates of CSF (see above) were either used directly for MS analysis of Ng, or subjected to proteolytic digestion. Samples selected for digestion were reconstituted in 10 µL 50 mM ammonium bicarbonate (Cat# 09830; Sigma‐Aldrich), shaken for 30 min and subjected to reduction by addition of 10 µL 10 mM DTT (30 min, 60°C). After cooling to 20‐25°C; 30 min), 5 µL 10 mM iodoacetamide (Cat# I6125; Sigma‐Aldrich) was added for alkylation (30 min at 20‐25°C, in dark). Thereafter, digestion was performed by adding 5 µL (25 ng) trypsin (Cat# V5111; Promega) at 37°C, over‐night; all carried out in 50 mM ammonium bicarbonate. All samples were dried under a vacuum centrifuge and subsequently stored at −20°C pending liquid chromatography‐tandem mass spectrometry (LC‐MS/MS) analysis.

Nanoflow LC coupled to electrospray ionization (ESI) hybrid quadrupole–orbitrap MS/MS (Dionex Ultimate 3,000 system and Q Exactive, both Thermo Fisher Scientific) was performed in a similar way as described previously (Brinkmalm et al., 2012; Gkanatsiou et al., 2019). Briefly, samples were reconstituted in 7 µL 8% FA/8% acetonitrile in water (v/v/v). An Acclaim PepMap 100 C18 trap column (20 mm × 75 μm; particle size: 3 μm; pore size: 100Å) was used for online desalting, and a reversed‐phase Acclaim PepMap RSLC column (150 mm × 75 μm; particle size: 2 μm; pore size: 100 Å) was used for separation (both Thermo Fisher Scientific). Mobile phases were 0.1% FA in water (v/v) (A) and 0.1% FA/84% acetonitrile in water (v/v/v) (B). Separation was performed at 300 nL/min flow rate by applying a linear gradient of 3% to 40% B for 50 min at 60°C. The mass spectrometer was operated in positive ion mode and the acquisition mass‐to‐charge (m/z) range was 350–1,800. Both MS and MS/MS acquisitions were obtained at a resolution setting of 70,000. MS/MS acquisitions were obtained using a normalized collision energy setting of 25, target values of 10^6^, and maximum injection time of 250 ms. Database search and semi‐quantitative analysis was performed with PEAKS Studio 8.5 and X+ (Bioinformatics Solutions Inc.) against a custom made neurogranin database and against the Uniprot human database. All fragment mass spectra were also evaluated manually.

### 2.9 Immunodepletion

For Ng immunodepletion, Ng monoclonal antibody (NG‐H6; Cat# sc‐514922; Santa Cruz Biotechnology) and for control, an in‐house neurofilament light monoclonal antibody (NFL‐21) was used. NG‐H6 or NFL‐21 antibodies were bound separately to each 1,500 μL Dynabeads™ M‐280 (Sheep Anti‐Mouse IgG; Cat#11202D, RRID:AB_2783009; ThermoFisher Scientific). In brief, the beads were initially washed twice in PBS using a magnetic particle concentrator. PBS‐washed aliquots of the bead suspensions were incubated for 1.5 hr with antibodies (3 μg antibody per 100 μL original bead suspension) followed by washing three times in PBS. Thereafter, the antibody beads were cross‐linked by resuspension in 20 mM of DMP (Cat# 21666; ThermoFisher Scientific) in 0.2 M triethanolamine (pH 8.2; Cat #90279; Merck), followed by incubation on a rocking platform for 30 min at 20‐25°C. Thereafter, the reaction was quenched by the addition of 1 M Tris HCl (Trizma Base, pH 7.5, Cat# T2319; Merck) to 50 mM and further incubation for 15 min at 20‐25°C. The cross‐linked antibody beads were then washed with PBS‐T and blocked with 10‐fold diluted Roti‐block (Cat# #A151.1; Carl Roth). The cross‐linked and blocked beads were washed twice with PBS and finally resuspended in PBS (1.5‐fold the original bead volume). Of this final bead suspension, 75 μL was added to a mixture of 100 μL CSF, 0.9 μL 10% TX‐100 and 7.3 μL complete protease inhibitor cocktail (25×; Cat# 4693116001; Merck) and incubated over‐night at 4°C. In addition to these incubations containing high concentrations of antibody‐coupled beads (samples labeled “high”), analogous incubations were set up containing added beads at 5‐fold lower bead concentrations (samples “medium”) and 25‐fold lower concentrations (samples “low”), and containing no beads (only PBS, samples “PBS control”). The incubation mixes were transferred to a KingFisher™ Flex System (ThermoFisher Scientific) to remove bead particles from the incubation mixes. Supernatant aliquots (150 μL) of the immunodepleted sample mixes were then analyzed for Ng using an enzyme‐linked immunosorbent assay (ELISA) with NG36 capture and NG2 reporter antibodies. This experiment was blinded for the experimenter since the samples were randomly distributed on a 96‐well block and the key was not shared with the experimenter.

### 2.10 Enzyme‐linked immunosorbent assay

Ng concentrations in the CSF immunodepletion experiment were measured using an in‐house sandwich ELISA, as described previously (Nazir et al., 2018). Briefly, NG36, a monoclonal antibody to the C‐terminal region of Ng, was used as a capture antibody, coated on Nunc Maxisorp 96‐well microtiter plates at 0.5 μg/mL in 50 mM sodium bicarbonate buffer (pH 9.6; 100 μL/well); the microtiter plates were incubated covered overnight at 4°C. The plates were then washed with PBS‐T (all washes were performed in PBS‐T). Thereafter, the unbound protein‐binding sites were blocked with 1% BSA in PBS (250 μL/well) by incubation on a shaker for 1 hr at 20‐25°C. The plates were washed (3 × 5 min) with PBS‐T and the calibrators (full‐length Ng with concentrations ranging from 7.8 pg/mL to 1,000 pg/mL), blanks, quality control and CSF samples (100 μL/well) were added to plates, and the plates were incubated for 1 hr at 20‐25°C. After another round of washing, the plates were incubated with biotinylated NG2 monoclonal detection antibody at 0.5 μg/mL (in PBS‐T with 1% BSA) and incubated for 1 hr at 20‐25°C. After washing, 100 μL/well enhanced streptavidin‐HRP (Cat# #4740 N; Kem En Tech), diluted 1:20,000 in PBS‐T with 1% BSA, was added to the plates and incubated for 30 min  at 20‐25°C. The plates were washed three times, followed by the addition of 100 μL of 3,3′,5,5′‐tetramethylbenzidine (TMB; Cat# 4380A; Kem En Tech) and incubated for 20 min at 20‐25°C) in the dark. The reaction was terminated by the addition of 100  μL of 0.2 M H_2_SO_4_ and, thereafter, the absorbance was measured at 450 nm (reference wavelength 650 nm) using an ELISA plate reader (Sunrise, Tecan Trading AG, Switzerland). Raw values obtained from ELISA for individual CSF samples were converted to pg/mL Ng (using the on‐plate calibrator values). The ELISA experiment was blinded because the samples were distributed randomly on the plate and the sample key was not shared with the experimenter. The study was exploratory, and no exclusion criteria were pre‐determined. The test for outliers was not conducted and therefore no data were excluded during analysis. Custom‐made material will be shared upon reasonable request.

### 2.11 Binding test for Ng and its peptides to heparin

Heparin beads were obtained from HiTrap heparin HP gel columns (GE healthcare). A 1 mL HiTrap heparin HP column was opened, the gel removed and resuspended in 10 mL binding buffer (10 mM sodium phosphate, pH 7). After 5 min centrifugation at 500 *g*, the soft gel was distributed into two Eppendorf tubes and centrifuged at 2000 *g* for 10 min. The soft gel pellet was finally resuspended in 1,500 μL binding buffer. For a binding test, 20 μL of the resuspended gel was used.

Samples of 2 μg of synthetic and recombinant Ng1‐78, or of 4 µg synthetic peptides Ng1‐42, Ng43‐78, Ng18‐42, Ng 21‐45, or Ng24‐48 in 50 μL binding buffer were incubated in Eppendorf tubes with 20 μL of either binding buffer or washed heparin beads (see above). The tubes were shaken at 100 rpm for 1 hr at 20‐25°C (Vortemp 56 shaker). Samples of 20 μL of the supernatants were taken and analyzed by SDS‐PAGE (4%–12%; reducing conditions). Bands were revealed with silver stain (PierceTM Silver Stain Kit; Cat# 24612; ThermoFisher Scientific). However, the silver stain did not stain the fragment Ng43‐78, most likely because of its positive charge at this pH (calculated pI: 11.6). Weak staining of a Ng27‐75 fragment has also been previously observed in an assay comparison study (Willemse et al., 2018). We therefore stained the gel subsequently also with the anionic stain Coomassie (Simply Blue stain; Cat# LC6065; ThermoFisher Scientific) to reveal also this fragment Ng43‐78. This experiment was not blinded for the experimenter.

### 2.12 Statistical analysis

Normality tests using Shapiro–Wilk demonstrated that the data were normally distributed for all groups (*p* > .05). No sample calculation was performed. For statistical analysis, unpaired *t*‐test with Welsh correction was used to find the statistical significance between the differences of control (PBS instead of beads) and immunodepleted samples (by NG‐H6 mab). Mean values for Ng remaining after depletion with NG‐H6 were compared using one‐way analysis of variance (ANOVA) followed by Tukey post‐hoc analysis. Statistical significance was defined as *p* < .05 *, *p* < .01 ** and *p* < .001 ***. Statistical analyses were performed either using SPSS (IBM SPSS Statistics for Windows, Version 22.0. Armonk, NY: IBM Corp) or Graphpad Prism 7 (GraphPad Software, Inc.).

## 3 RESULTS

### 3.1 Neurogranin is present in different molecular forms in CSF

**FIGURE 2:**
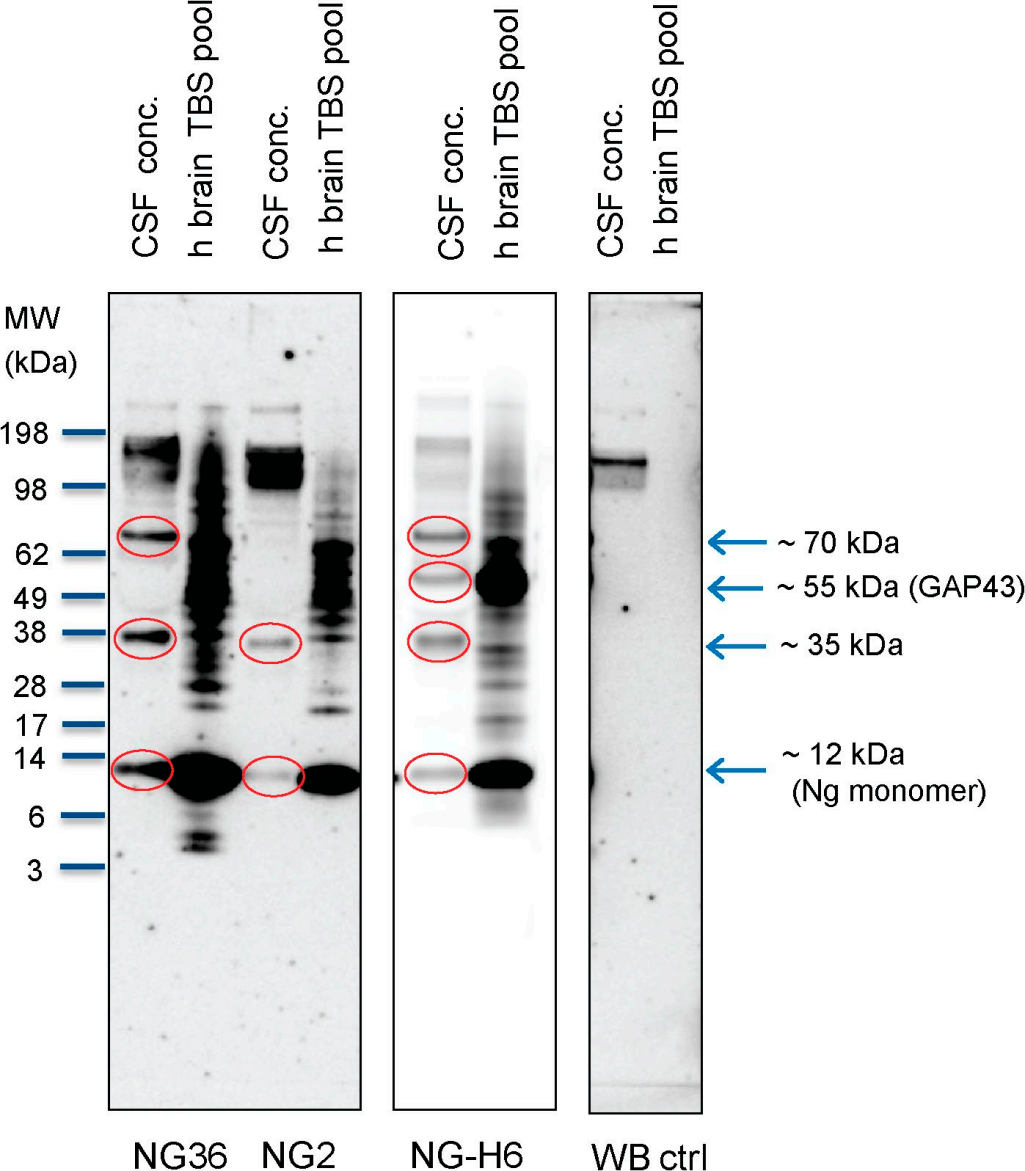
Ng is present in CSF as full‐length monomer and as higher molecular weight species. The panels represent immunoblots of pooled CSF concentrates and pooled TBS extracts from human brains (as Ng standard). Human brain extact (“h brain TBS pool”) was used as a positive control for the presence of monomeric Ng. Samples were analyzed on 4%–12% SDS‐PAGE gels at non‐reducing conditions. NG36, NG2 and NG‐H6 (below panels) were the mabs used to detect Ng species. Red circles denote the major bands detected and the arrows indicate their apparent molecular sizes: Ng monomer at approx. 12 kDa, and higher molecular weight species at approx. 35 kDa and 70 kDa. The band at 55 kDa in the NG‐H6 immunoblot most likely represents GAP43, which is known to cross‐react with the NG‐H6 antibody, since that antibody recognizes an epitope in the IQ domain which is present both in Ng and GAP43 (see also Figure S1). WB ctrl, western blot control probed with mouse IgGκ‐binding protein‐HRP only

Previous reports have shown the presence of Ng fragments in CSF. The majority of those fragments begin at aa 43, indicating that this is a major cleavage site of Ng in CSF. This site is located in the functionally important IQ domain (see Figure 1). In an effort to detect the molecular forms of Ng appearing in CSF, we analyzed pooled concentrated CSF samples on immunoblots (see Figure 2). We used two in‐house generated monoclonal antibodies (mab), namely NG36 and NG2, with estimated epitopes of Ng63‐77 and Ng52‐63, respectively (Becker et al., 2018; Kvartsberg, Duits, et al., 2015) and the commercial NG‐H6 monoclonal antibody with the previously mapped epitope estimated around Ng37‐39 (Höglund et al., 2020). All the above‐mentioned antibodies reacted with monomeric Ng (~ 12 kDa) in CSF; that band co‐migrated with the Ng band from human brain. Furthermore, all these antibodies reacted with Ng bands around 35–38 kDa in CSF (further on called “~ 35 kDa” band). In addition to the immunoreactive bands mentioned, NG36 and NG‐H6 reacted with Ng bands around 70 kDa in CSF. Furthermore, NG‐H6 cross‐reacted with a band of around 55 kDa in CSF; however, the other monoclonal antibodies (NG2 and NG36) did not produce any immunoreactive bands corresponding to this molecular weight. It has been suggested that NG‐H6 cross‐reacts with the IQ domain which is present in both Ng and in growth‐associated protein 43 (GAP43) (Höglund et al., 2020). We have used GAP‐43 monoclonal as well as polyclonal antibodies to show that GAP‐43 is indeed detected at 55 kDa in TBS brain extracts (Figure S1) ; therefore, the band detected around 55 kDa with NG‐H6 very likely represents GAP43 (Figure 2). All those immunoreactive bands were not detected in control immunoblots where only mouse IgGκ‐binding protein‐HRP‐conjugate was used. This clearly suggests that Ng is present in several molecular forms in CSF. Among these anti‐Ng monoclonal antibodies, NG36 produced more intense Ng‐immunoreactive bands as compared to NG2 and NG‐H6, when the samples were probed with similar antibody concentrations. Therefore, we chose NG36 antibody to achieve the highest sensitivity in the following western blots.

**FIGURE 3:**
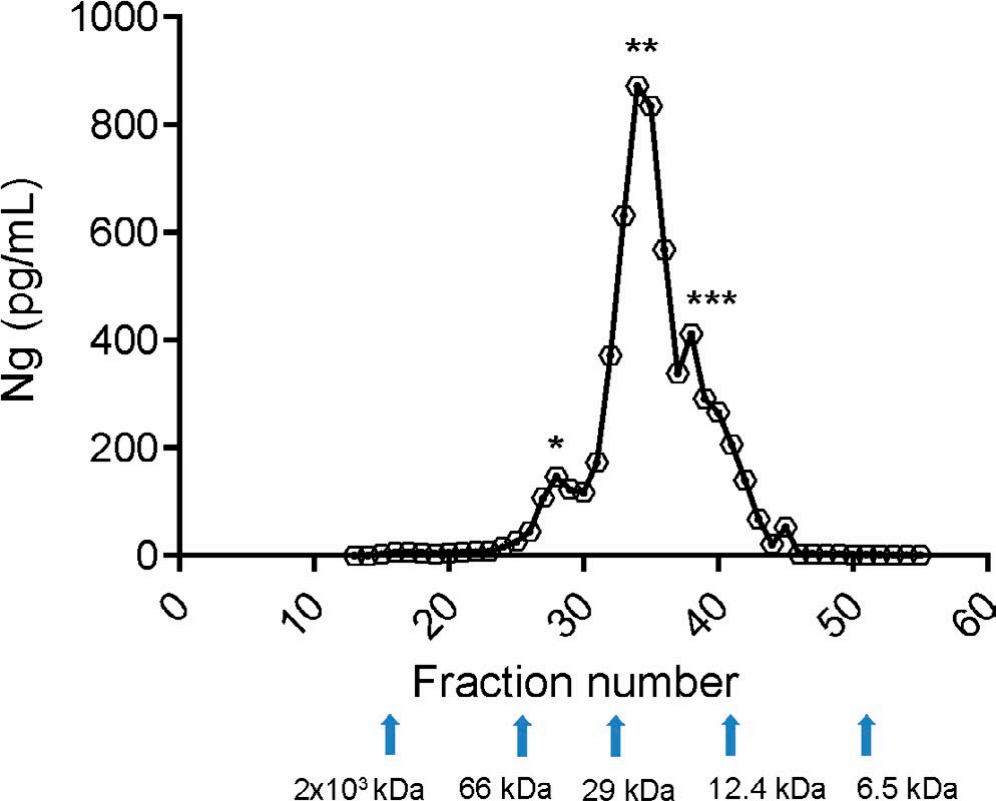
CSF Ng is present in various fractions after size exclusion chromatography (SEC). Pooled CSF was concentrated by ultrafiltration, followed by SEC. The elution positions of SEC size markers (in kDa) are indicated by arrows below the graph. **, major peak at ~25 kDa, which is close to the reported size range (~22 kDa) of monomeric Ng in Triton X‐100 containing buffer (Kumar et al., 2013). * and ***, minor peaks, before and after the main peak containing higher and lower molecular weight species, respectively

For a more detailed analysis of the molecular species contained in CSF, a larger pool of CSF was concentrated by ultrafiltration using a low molecular cut‐off filter membrane (3 kDa) and subjected to SEC with fraction collection. All the fractions were analyzed for Ng using an in‐house ELISA. This initial experiment suggested that Ng is present in various fractions eluting at different molecular weights (Figure 3). We found that monomeric Ng was eluted at ~25 kDa (as determined by SEC size standards) as the major peak. Minor peaks before and after that major peak indicated the presence of higher and lower molecular weight forms of Ng, respectively. To consolidate this initial finding, we concentrated other pools of CSF and repeated the same procedure, described in sections 2.3 and 2.4, and we performed western blotting of the SEC fractions to detect the molecular forms of Ng and to determine their apparent molecular weights also by SDS‐PAGE molecular weight standards.

### 3.2 Detection of Ng forms containing the C‐terminal region

**FIGURE 4:**
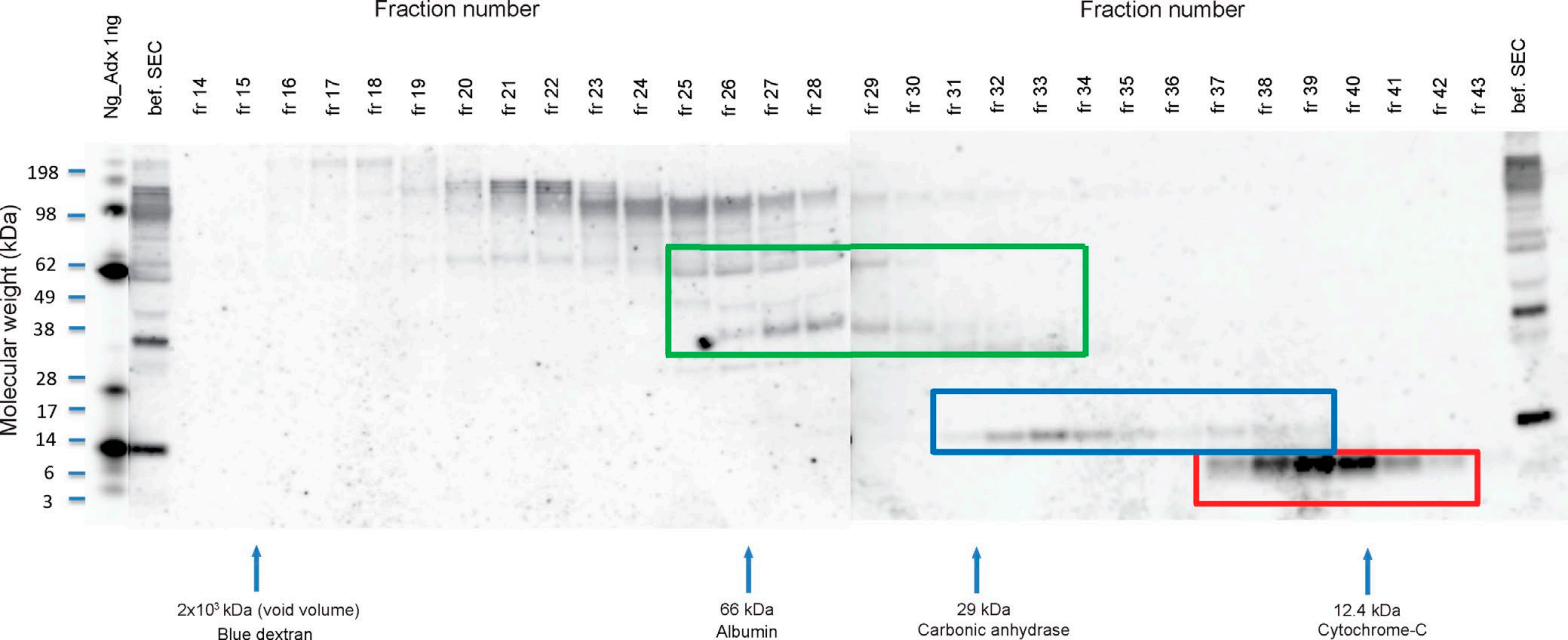
Neurogranin is present in different molecular forms in CSF. Size exclusion chromatography (SEC) fractions (fractions 14–43; on adjacent blots) were collected and processed for SDS‐PAGE at non‐reducing conditions followed by immunoblotting (mab NG36). The positions of SDS‐PAGE size markers are shown on the left side and the fraction numbers are shown above each lane on the blots. The SEC column was initially calibrated with SEC size markers; their elution positions are indicated by arrows below the blots. Ng_Adx, synthetic Ng1‐78 (as positive control). This lane is shown here at lower exposure than the rest of the blot to avoid overexposure of the bands. Fraction “bef. SEC” represents the concentrated CSF sample before SEC, but 2.5x less volume of this sample was used for blotting, as compared to the samples from the column fractions. The rectangle shown in green corresponds to molecular weight species of Ng above 12 kDa, the rectangle shown in blue corresponds to ~12 kDa and the rectangle shown in red corresponds to Ng species of ~6 kDa

After concentration and SEC, followed by PAGE of the fractions under non‐reducing conditions and western blotting using the NG36 monoclonal antibody, bands of around 12 kDa (blue rectangle in Figure 4) and ~6 kDa (red rectangle in Figure 4) were detected, probably corresponding to monomeric Ng and C‐terminal Ng‐fragments, respectively. In addition, ~35 kDa and ~70 kDa bands were detected (green rectangle in Figure 4), corresponding to higher molecular weights as compared to monomeric Ng. Figure 2 had shown previously that the 70 kDa bands reacted also with antibody NG‐H6 (detecting Ng N‐terminally of Ng aa43), whereas the 35 kDa band reacted additionally with both antibody NG‐H6 and antibody NG2 (detecting C‐terminal Ng). The corresponding control blots (Figure S2) show the lack of such bands in the absence of primary antibody). We detected also faint bands around 30 kDa, but these were also present on the control blot (Figure S2) and were therefore considered unspecific. The immunoblotting performed under reducing conditions using another pool of CSF is shown in Figure S3. The monomeric Ng band as well as a weak band at 6 kDa were detected. However, no specific higher molecular weight bands (relative to monomeric Ng) were observed, indicating that the higher molecular weight forms of Ng were likely disulfide‐linked complexes because of their sensitivity to reduction.

To allow for a more stringent identification of Ng peptides in CSF, the concentration and SEC separation was repeated. Again, fractions representing molecular forms of Ng at around 35 kDa and 70 kDa, and those representing monomeric Ng at ~12 kDa were identified. However, the fractions representing the smaller than monomeric form of Ng (at around 6 kDa) were much weaker this time; the reason for this is currently not known, but it may have to do with the use of a different CSF pool, changed adsorption of peptides on the SEC column, or with unreliable detection of such small fragments on nitrocellulose blots. Although we cannot make quantitative comparisons of the different Ng species at this time, the main outcome of the SEC experiment is that there is now evidence of the presence of such molecules in CSF.

**FIGURE 5:**
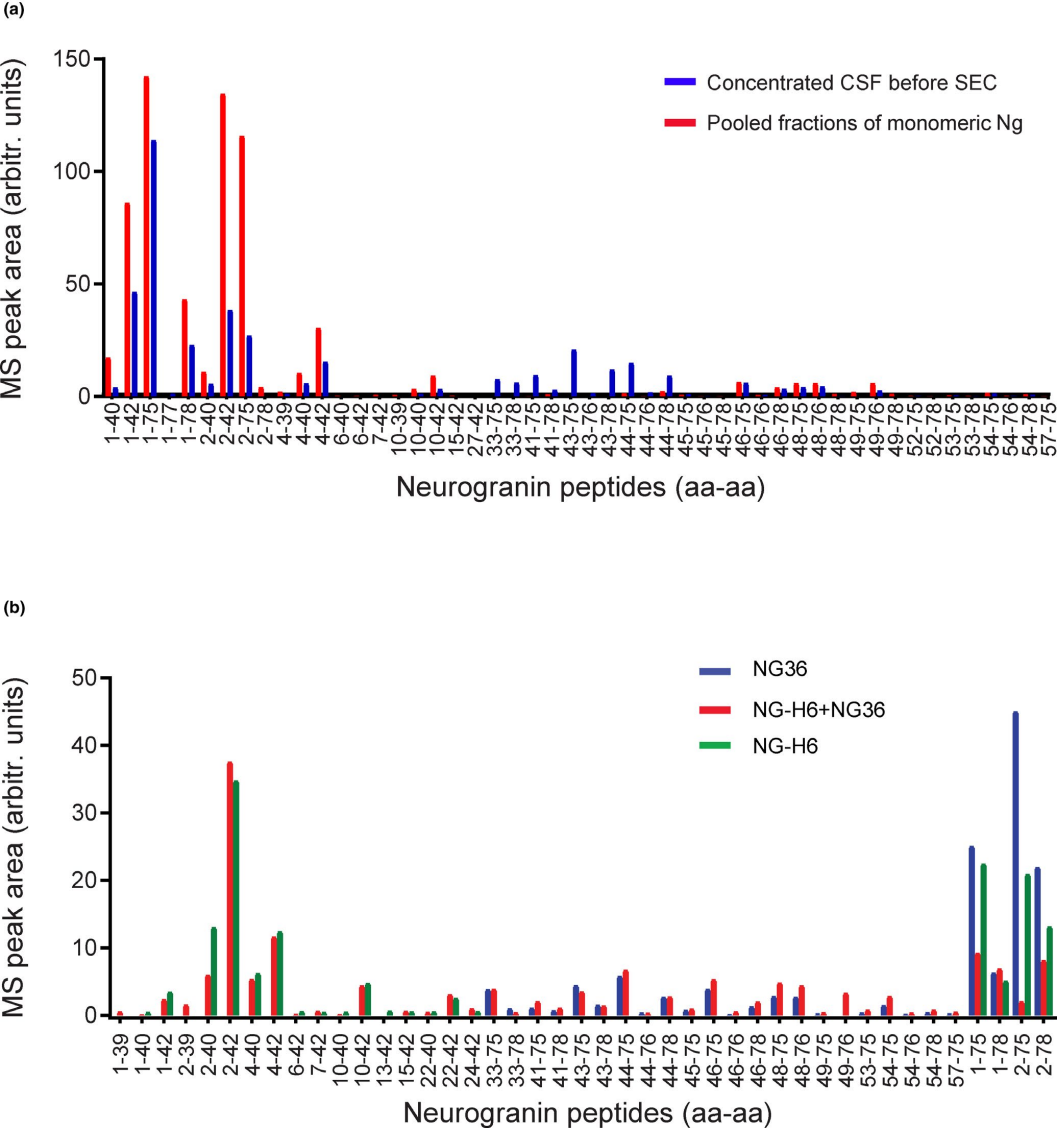
Neurogranin peptides identified by MS/MS in immunoprecipitates. (a) A pool of CSF was concentrated by ultrafiltration and then size‐separated on a SEC column. Ng peptides were immunoprecipitated from the concentrated CSF pool before (blue bars) and after SEC separation (red bars) by a mixture of bead‐immobilized mabs NG36 and NG‐H6. (b) Neurogranin peptides identified in the non‐concentrated CSF pool [same pool as in (a)] by NG36, NG‐H6, and a mixture of NG36 and NG‐H6. Only peptides which had at least one peak area (among the three immunoprecipitations) of more than 1% of the largest peak area are shown. The heights of the bars in (a) and (b) reflect peak areas of identified peptides (sum of all major known modifications for each peptide)

To get more details about those molecular forms, fractions containing the 35 kDa and 70 kDa bands, and those containing the 12 kDa band were each pooled and analyzed by immunoprecipitation, followed by mass spectrometry, using a mixture of immobilized mabs NG‐H6 and NG36 to bind Ng peptides both N‐terminal and C‐terminal of the Ng42/43 calpain‐1 cleavage site (Becker et al., 2018), as well as full‐length Ng. The peptides identified are summarized in Figure 5a. Ng was only found in the pooled fractions representing the 12 kDa band; it was not possible to identify any Ng peptides in the pooled fractions representing the 35 kDa and 70 kDa band, most likely because of low abundance and/or low sensitivity of MS at this mass range. The peptides found in the 12 kDa pool covered the whole range of the Ng sequence, N‐terminal and C‐terminal peptides and full‐length Ng. Interestingly; we found several dominant peptides starting at aa 2. The significance of that cleavage site is still unknown. The mass spectrometric peak areas representing N‐terminal peptides dominated, however this may be a consequence of the very potent binding properties of the mab NG‐H6. Because small peptides may have been lost during the concentration of the CSF pool, we repeated this immunoprecipitation experiment using a non‐concentrated pool. In this case, we separately immobilized NG36 and NG‐H6 on beads, and used these and a mixture of both in the immunoprecipitation experiment. As can be seen in Figure 5b, NG‐H6 bound to N‐terminal peptides NgX…42 ("X" denoting undefined amino acid in sequence) and full‐length Ng, whereas NG36 bound to C‐terminal peptides starting at Ng33…75 and full‐length Ng. Again, strong signals were found for Ng peptides starting at aa 2 and ending at aa 75, as well as for full‐length Ng.

As mentioned earlier, the lack of identified Ng peptides in the 35 kDa and 70 kDa fractions may have been because of the low sensitivity of detection by MS for peptides in this high mass range. Therefore, in another SEC separation of CSF, the corresponding fractions were analyzed again by immunoprecipitation (NG36 antibody) and MS, but with prior trypsinization of the eluates. This time, tryptic C‐terminal peptides Ng54‐68 and Ng55‐68 were identified in the monomeric Ng fractions, whereas the 35 kDa and 70 kDa fractions contained only Ng54‐68 at very low concentrations (together ~1% of that present in monomeric fractions). In the fractions corresponding to the 35 kDa protein band, a search of the peptides against the Uniprot human database showed only one protein other than Ng, prostaglandin‐H2 D‐isomerase (PTGDS; Uniprot P41222) to be found in the NG36 immunoprecipitate but not in the control immunoprecipitate (24% coverage; 4 unique peptides versus 0 peptides).

### 3.3 Ratio of C‐terminal peptides to total neurogranin in CSF

A large number of C‐terminal fragments of Ng had been previously identified in CSF using immunoprecipitation followed by matrix‐assisted laser desorption/ionization (MALDI) MS (Kvartsberg, Duits, et al., 2015; Kvartsberg, Portelius, et al., 2015), most of them starting C‐terminally of the main calpain‐1 cleavage site Ng42/43 on Ng (Becker et al., 2018). Our results (Figure 5a and b) show that there are also significant amounts of N‐terminal Ng fragments present in CSF, most of them ending in the same region, near Ng42/43. Therefore, it appears as if those N‐ and C‐terminal fragments have been generated by cleavage of full‐length Ng by calpain‐1. However, there is also full‐length Ng present in CSF [(Thorsell et al., 2010) and Figure 5a and b]. It would be of interest to know whether the ratio of fragments to full‐length Ng increases in CSF during progression to AD. Such knowledge could give hints to proteolytic events involved in AD pathogenesis or it may be useful for the development of biomarkers. A shift from full‐length Ng to Ng fragments in the disease progression may also be of relevance for the choice of an ELISA assay targeting either full‐length Ng, or full‐length and Ng fragments. Therefore, we set out to estimate the degree of cleavage of Ng in CSF samples from control and AD individuals using our in‐house ELISA which is based on two C‐terminal Ng antibodies. In analogy, we could have also used an N‐terminal ELISA for this purpose, but such one was not readily available.

**FIGURE 6:**
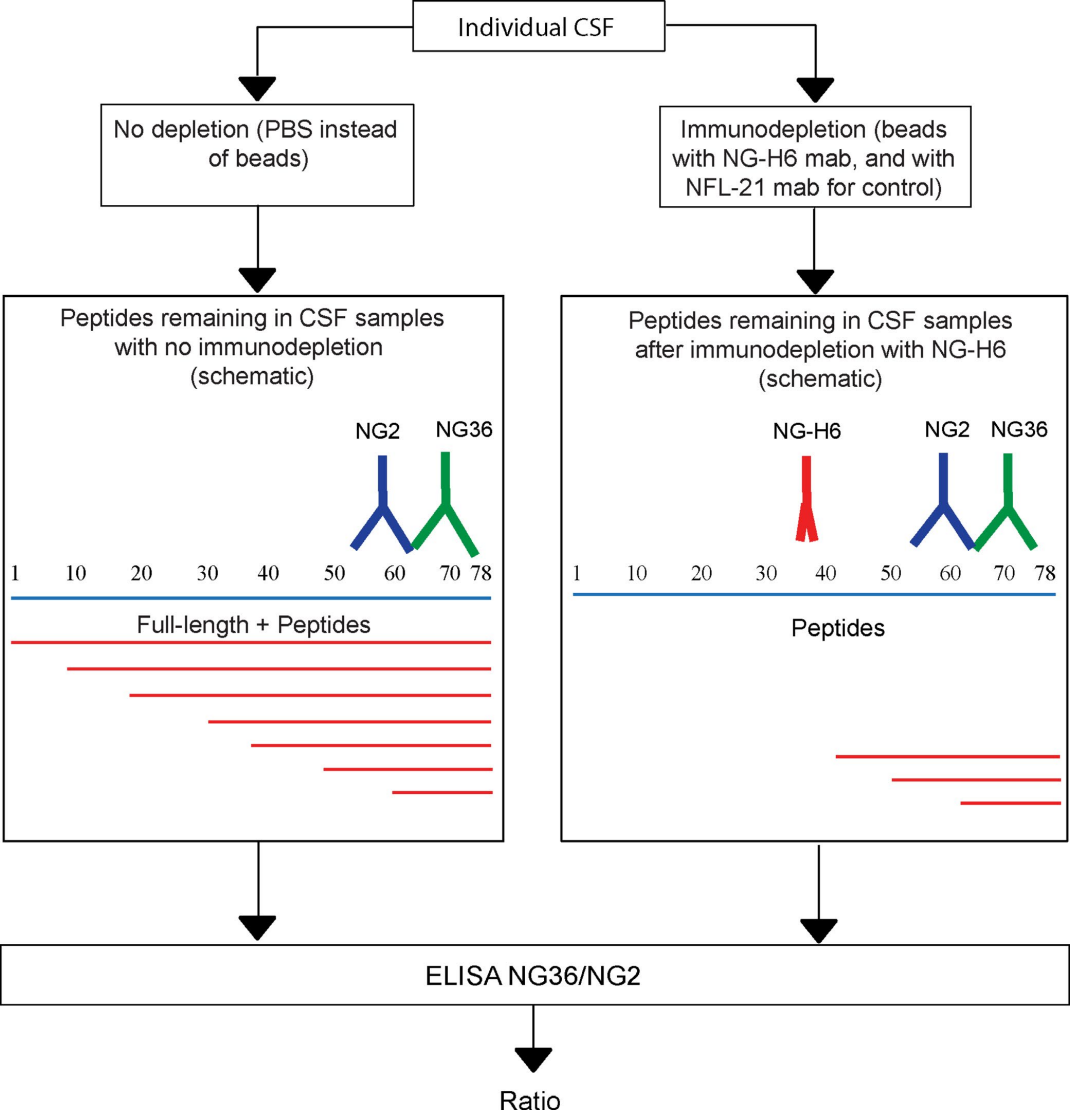
Schematic representation of the workflow to analyze the proportion of C‐terminal Ng fragments to “total‐Ng”. Total‐Ng comprises all Ng species detectable by the C‐terminal sandwich ELISA used (= C‐terminal fragments and full‐length Ng). Individual CSF samples were either immunodepleted of N‐terminal and full‐length Ng using NG‐H6 beads, or using NFL‐21 control beads, followed by analysis of the supernatants using an in‐house sandwich ELISA with NG36 as capture antibody and NG2 as the detection antibody

**FIGURE 7:**
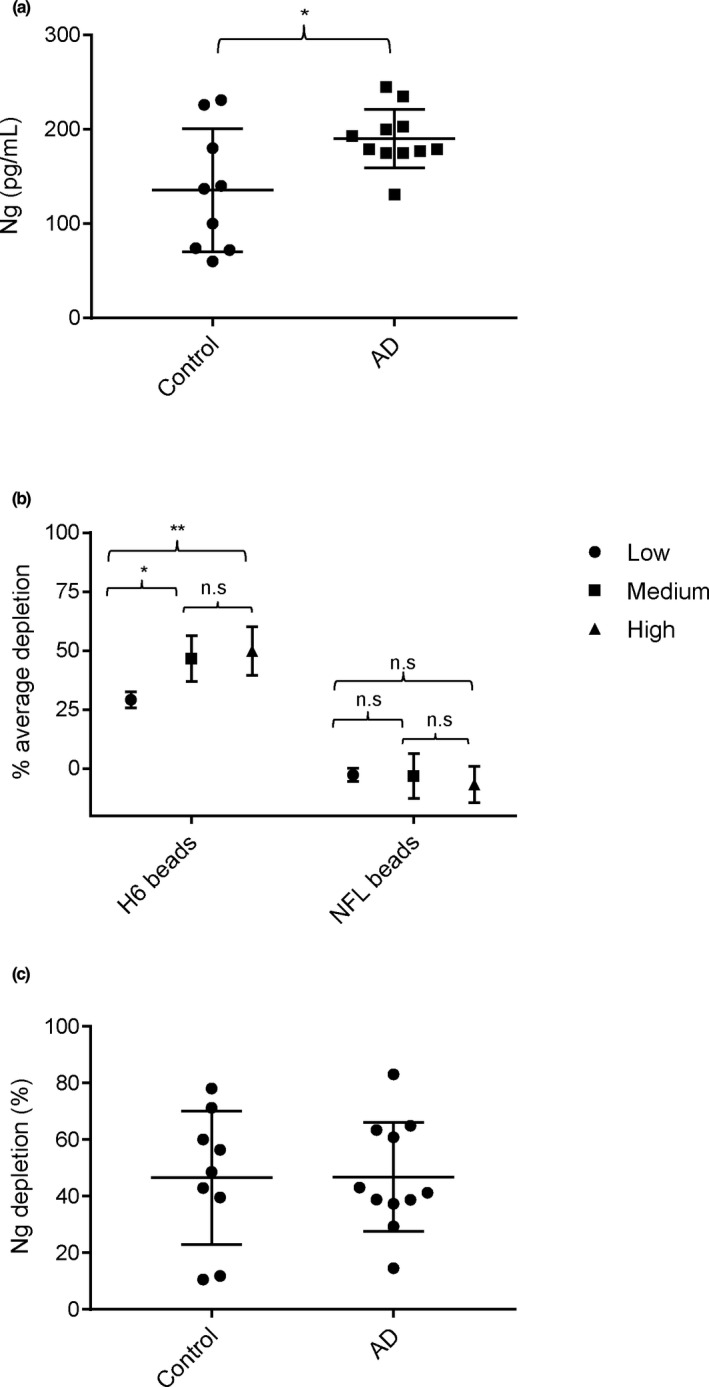
Immunodepletion of full‐length Ng and Ng fragments (starting N‐terminally of ~aa 43) from CSF using NG‐H6 beads. (a) Levels of Ng in control and AD samples before immunodepletion by sandwich ELISA; *p* = .04 (unpaired *t*‐test with Welsh's correction); N, number of individuals: control = 9 and AD = 11; median control = 137, median AD = 179; control *SD* = 65.3, AD *SD* = 31.1. The mean and standard error of mean (*SEM*) are indicated in the scatter plot. (b) Depletion levels reach saturated maximum levels. NG‐H6 and NFL‐21 (control) mab were cross‐linked separately to beads. Those beads were then diluted with PBS to three concentrations of beads (high, medium, low; five‐fold dilution steps) and used at equal bead suspension volumes to deplete Ng species from CSF. Average depletion (all samples) reached a plateau at middle and high bead concentrations of NG‐H6 beads, but not at low bead concentration. NFL control beads did not show depletion. One‐way ANOVA followed by Tukey's post‐hoc analysis shows the significant Ng reduction when compared between low and medium NG‐H6 antibody bead concentration and between low and high NG‐H6 bead concentration. No significant (n.s) differences were observed between medium and high NG‐H6 bead concentration; the immunodepletion had reached saturation at these concentrations. No depletion of Ng was detected with NFL‐21 antibody at any NFL‐21 bead concentration (high, medium, and low) and therefore no significant differences were found using one‐way ANOVA followed by Tukey's post‐hoc analysis. The data points represent the percentage of Ng depleted (0% depletion = no beads but PBS added); the error bars indicate *SEM*, *n* = 20 for each concentration of beads, *p* < .05 *, *p* < .01**. NG‐H6 mab depletes Ng significantly (unpaired *t*‐test) at all bead concentrations used, as compared to NFL‐21 antibody (NG‐H6 low versus NFL‐21 low, *p* < .001; NG‐H6 medium versus NFL‐21 medium, *p* < .001; NG‐H6 high versus NFL‐21 high, *p* < .001. (c) Ng depletion (%) in control and AD samples after immunodepletion with NG‐H6 beads using medium bead concentration; *p* = .97 (unpaired *t*‐test with Welsh's correction); N, number of individuals: control = 9 and AD = 11; median control = 48.5, median AD = 41.2; control *SD* = 23.58 and AD *SD* = 19.3. The mean and standard error of mean (*SEM*) are indicated in the scatter plot

However, such C‐terminal ELISA assays (De Vos et al., 2015; Kvartsberg, Duits, et al., 2015) are unable to distinguish between the signal contribution from full‐length Ng and C‐terminal Ng peptides because they measure “total‐Ng” (all Ng species containing the epitopes of both C‐terminal antibodies of the ELISA sandwich assay). Therefore, we applied a strategy to remove full‐length Ng and near full‐length Ng fragments from CSF samples by immunodepletion (Figure 6). As the antibody for immunodepletion, we used the NG‐H6 antibody, which binds to the Ng IQ domain at approximately aa 37–39 and recognizes Ng1‐42, but not more C‐terminal fragments Ng43…X ("X" denoting undefined amino acid in sequence) (Höglund et al., 2020). The immunodepleted samples thus contain only fragments of approximately Ng43…X (Figure 6), which can be quantified by a C‐terminal Ng sandwich ELISA (NG36/NG2; see Figure 1). The ELISA signal obtained after immunodepletion with the NG‐H6 antibody beads is then compared to the signal from an undepleted CSF aliquot of the same sample, which therefore contains “total‐Ng” (C‐terminal fragments, longer N‐terminal fragments, and full‐length Ng; see Figure 6). The samples representative for control and AD individuals were selected on the basis on AD biomarkers (as explained in section 2.2). The age, total‐tau, phospho‐tau, and amyloid‐beta concentrations for these individuals are summarized in Table 1. Figure 7a shows the Ng levels of control versus AD CSF samples, as determined by the C‐terminal Ng sandwich ELISA (NG36/NG2) [*p* = .04 (unpaired *t*‐test with Welsh's correction), N, number of individuals: control = 9 and AD = 11; median control = 137, median AD = 179; control standard deviation (*SD*) = 65.3, AD *SD* = 31.1].

As a control for non‐specific depletion of Ng ELISA signal by IgG beads themselves, control beads carrying NFL IgG antibody at the same concentrations were used. To be able to compare the signals before and after depletion (to determine the ratio), an aliquot of the sample was diluted with the same volume of PBS as compared to the bead suspension. Three concentrations of beads were used to estimate if saturation levels of depletion were achieved. Figure 7b shows that depletion levels of about 50% were obtained with NG‐H6 beads at the highest concentration of beads. Almost the same depletion level (and not significantly different), ~50% [For middle bead concentration: NG‐H6 *SD* 20.7 versus NFL‐21 (control) *SD* 20.1], was reached using a five times lower bead concentration, thus indicating near‐complete removal of full‐length Ng and fragments carrying the NG‐H6 antibody epitope even at this lower bead concentration. The level of around 50% can therefore be considered the maximum level of depletion. The lowest bead concentration (five times lower than the middle concentration) did not achieve complete depletion, namely only 29%. The NFL‐21 control beads did not significantly deplete the Ng signal at all bead concentrations.

The results for the samples grouped into “control” and “AD‐like” are shown in Figure 7c. Both control and AD‐groups had about the same ratio of C‐terminal Ng fragments to total‐Ng of about 50%, with no significant difference between the two groups. These results show that there was a similar processing in both groups of control and AD samples, indicating that the overall pattern did not change in progression to AD [*p* = .97 (unpaired *t*‐test with Welsh's correction); N, number of individuals; control = 9 and AD = 11; median control = 48.5, median AD = 41.2; control *SD* = 23.58 and AD *SD* = 19.3]. Given the high variability seen in Figure 7c in both groups of samples, it seems unlikely that this method would be able to detect quantitative changes of subgroups of Ng peptides [e.g., the ones reported in (Kvartsberg et al., 2019)]. To profile changes for individual peptides, more selective methods of quantitation will therefore be required (e.g., IP‐MS; see also discussion).

### 3.4 Ng protein and Ng fragments containing KKIK sequence bind to heparin

**FIGURE 8:**
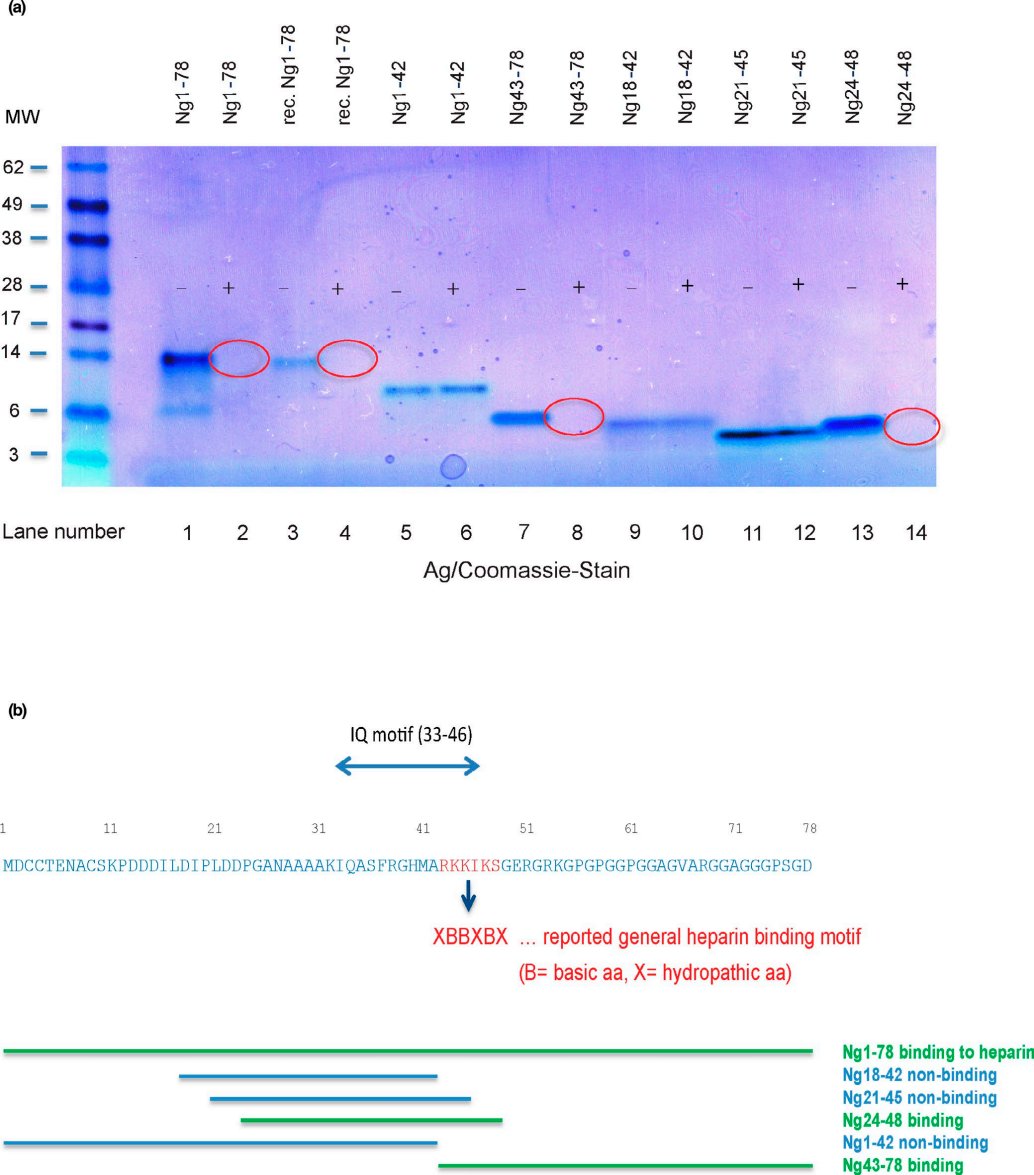
(a) Full‐length Ng and C‐terminal Ng peptides containing KKIK aa sequence motif, but not N‐terminal peptides NgX…42 ("X" denoting undefined amino acid in sequence), bind to heparin beads. Synthetic and recombinant (rec.) Ng and Ng peptides, either exposed to PBS control (“‐”, in stained gel) or heparin beads (“+”), were separated under reducing conditions on a 4%–12% SDS PAGE gel. The gel was then sequentially stained by silver stain and Coomassie stain. The red circles point to the disappearance of the peptide bands because of binding of the peptides to heparin beads. MW, molecular weight markers (kDa). (b) Summary of the heparin‐binding properties of full‐length Ng and Ng peptides

The ability of full‐length Ng and various N‐terminal and C‐terminal fragments to bind to heparin beads was tested in vitro, as this may indicate whether Ng forms may be secreted from neurons via the non‐conventional transport mechanism (see discussion). For the binding experiment, agarose beads with immobilized heparin, or control buffer aliquots were incubated with each of various synthetic or recombinant Ng peptides. Analysis of the supernatants after separation of the beads revealed whether the Ng species had bound to the beads and were therefore depleted in the supernatants. Figure 8a shows the disappearance of Ng from the supernatants of full‐length Ng (compare lanes 2/1 and 4/3) and of C‐terminal fragments Ng43‐78 (lanes 8/7), Ng24‐48 (14/13); however, N‐terminal Ng peptides Ng1‐42, Ng18‐42, and Ng21‐45 did not bind. Figure 8b summarizes the location of the peptides along the Ng sequence. The binding pattern of the peptides suggests that indeed the sequence RKKIK contains the amino acid residues responsible for heparin‐bead binding (peptide Ng21‐45, which did not bind, carried only part of the presumed heparin‐binding motif).

## 4 DISCUSSION

Synaptic dysfunction and degeneration are important events during neurodegeneration in AD (Davidsson & Blennow, 1998; Maiti et al., 2015). Ng secretion into CSF is markedly increased in AD (Kvartsberg, Duits, et al., 2015; Thorsell et al., 2010).

A better knowledge of the forms of Ng that are present in CSF will be helpful in understanding which of the observed molecular species of Ng are targeted by the various types of Ng ELISA assays and which ones escape detection. In this study, we aimed at identifying molecular forms of Ng in CSF and at investigating whether there were differences in the proportion of C‐terminal peptides to total‐Ng during progression to AD.

This study, which used pools of CSF from anonymized clinical samples of CSF, suggests that there are several molecular forms of Ng present in CSF, some that have not previously been found in CSF (N‐terminal fragments, and higher molecular weight forms of Ng).

Full‐length or near to full‐length Ng is detectable at ~12 kDa, as suggested in several studies using reducing SDS‐PAGE (Becker et al., 2018; Thorsell et al., 2010). In this study, we used both reducing and non‐reducing SDS‐PAGE followed by immunoblotting using C‐terminal specific antibody (NG36, immunogen Ng63‐77). Under both reducing and non‐reducing conditions, we could clearly detect a band at ~12 kDa by direct immunoblotting of concentrates of CSF (Figure 4, in blue box). This band carried two distinctly located epitopes for Ng monoclonal antibodies, namely for NG36 and NG‐H6; because of its apparent MW, it can therefore be considered to represent full‐length or near‐full‐length monomeric Ng. This band co‐migrated with synthetic Ng1‐78 peptide (Figure 4) which also showed several higher molecular weight species in non‐reducing SDS‐PAGE western blots. This is the first report showing direct detection of both full‐length Ng as well as Ng C‐terminal fragments from CSF by western blotting. An earlier report (Thorsell et al., 2010) required prior enrichment by IP before immunoblot analysis to be able to detect full‐length Ng, and no fragments were detected. Several improvements may have led to the increase in detection sensitivity, especially the availability of antibodies with higher affinity, and the concentration of CSF sample prior to western blot by ultrafiltration, but likely also the fixation of blots after transfer to minimize losses of Ng and its fragments.

Several C‐terminal truncated peptides are present in CSF (Kvartsberg, Duits, et al., 2015). Furthermore, our previous finding suggested that calpain‐1 and prolyl endopeptidase (PREP) cleave Ng and generate several C‐terminal fragments that give bands on SDS‐PAGE at ~6 kDa (Becker et al., 2018). The occurrence of Ng in late fractions of SEC (fractions collected after monomeric Ng) and the detection of a band around 6 kDa using monoclonal C‐terminal specific antibody suggests that Ng C‐terminal peptides are present in CSF, which is in agreement with earlier MS‐based data (Kvartsberg, Duits, et al., 2015). Moreover, this is the first report of showing by IP‐MS the presence of significant amounts of N‐terminal Ng fragments, ending at or close to the dominant calpain‐1 cleavage site between aa 42 and 43. It appears that most of the N‐terminal and C‐terminal peptides were generated by cleavage at and close to the Ng42/43 calpain‐1 cleavage site (see also Figure 5a and b). In addition, we found significant amounts of full‐length Ng and near full‐length Ng, Ng2‐75. Ng peptides ending at −75 have been targeted using a sandwich ELISA (De Vos et al., 2016) and shown to be already elevated in CSF of patients with mild cognitive impairment (MCI) with a high probability for AD (MCI patients). Taken together, these new data open new avenues of research on N‐terminal peptides of neurogranin and on truncated peptides at Ng1_2 and Ng75_76 as potential additional AD biomarkers and for investigating the role of Ng's cleavages in the disease process of AD or in normal physiology.

Our SEC fractionation of concentrated CSF followed by ELISA analysis suggests the appearance of monomeric Ng in fractions corresponding to 25 kDa (by SEC standards) which is different from the apparent molecular weight as determined by western blotting. However, our results showing monomeric Ng eluting in SEC at 25 kDa are in line with a previous report (Kumar et al., 2013), which attributed the apparent about 3–4 times higher MW of Ng in SEC (compared to the theoretical MW) to the unfolded nature of Ng and therefore different hydrodynamic properties as compared to the more folded and compact standards used to calibrate the SEC column.

Besides monomeric Ng and C‐terminal Ng fragments, we also observed weak bands of higher MW than monomeric Ng. These bands, around ~35 kDa and ~70 kDa were detected using the NG36 monoclonal antibody in the non‐reduced samples of CSF. Similarly, trypsin‐digested eluates of NG36 antibody immunoprecipitates from the pooled fractions yielding these higher molecular weight bands revealed the presence of Ng peptide Ng54‐68 by MS, but at much less abundance than the monomer Ng fractions. This suggests that these molecular forms could either be full‐length oligomers of Ng (as observed in the peptide standard Ng1‐78 on non‐reducing western blot), or Ng complexed with other proteins. We were interested in the identity of such proteins as they could point to new interaction partners involved in neurogranin´s cellular function or have a role for Ng secretion. The intensity of these bands largely decreased for reduced samples which would indicate that these complexes are held together via disulfide bridges. The only protein candidate identified from the NG36 immunoprecipitate by MS was prostaglandin‐H2 D‐isomerase (PTGDS; uniprot P41222), but the significance of this initial finding is at the moment unclear.

The higher MW species of Ng are already visible by western blot in concentrated CSF prior to SEC fractionation and correspond to a peak in ELISA signal of SEC eluates prior to the main peak of monomeric Ng. Therefore, it is unlikely that complexes were artefactually formed during the sample preparation process for SDS‐PAGE, but were present in the concentrated CSF sample already.

The presence of C‐terminal fragments observed in this study and reported in previous studies from our group (Kvartsberg, Duits, et al., 2015) prompted us to identify the ratio of C‐terminal fragments to total‐Ng present in CSF. Immunodepletion using NG‐H6, which binds to full‐length Ng and N‐terminal Ng peptides, followed by an in‐house sandwich ELISA using NG36 and NG2 monoclonal C‐terminal specific antibodies suggested that on average ~50% of the peptides identified in the CSF samples were C‐terminal peptides. This gives a first indication why ELISA assays targeting full‐length Ng had a similar power for distinguishing AD from control samples as those assays targeting both full‐length Ng and its C‐terminal peptides (Willemse et al., 2018). An earlier report, studying TBS brain extracts by IP‐MS, found significant differences of several C‐terminal Ng peptides between control and AD samples (Kvartsberg et al., 2019). Our method, which measures the overall signals of Ng and Ng peptides by ELISA in CSF, could not find significant differences in the extent of depletion by NG‐H6 between control and AD‐like samples. However, different tissues were studied, and the peptides that showed the largest differences in the brain study constituted only a smaller percentage of the C‐terminal peptides measured by ELISA. Future studies by IP‐MS [as in (Kvartsberg et al., 2019)], aiming at analyzing changes of individual Ng peptides in CSF, may indeed find some peptides showing a large change, indicative of proteolytic events during AD progression.

Another report suggested that C‐terminal peptides are increased in CSF from AD patients as compared to controls (De Vos et al., 2015) possibly because of up‐regulation and activation of calpain‐1 in AD brain (Kurbatskaya et al., 2016). At the time of that study, it was not yet realized that near full‐length Ng comprises a large percentage of all Ng present in CSF. That study used mab NG7 (binding to Ng near the C‐terminal end) as a capture antibody, and the polyclonal antibody 07–425 (binding at Ng77‐78). Therefore, this assay measured the sum of C‐terminal fragments and full‐length Ng, and it cannot be determined by that method whether only C‐terminal fragments or also full‐length Ng was increased.

Whether C‐terminal Ng fragments have a specific function remains unknown. Our previous study indicated one of the main calpain cleavage site is between the 42nd and 43rd aa of its amino acid sequence generating N‐terminal and C‐terminal halves of Ng (Becker et al., 2018). Strikingly, full‐length Ng and its C‐terminal half carry the sequence RKKIK which may represent the heparin‐binding motif XBBXBX (Cardin & Weintraub, 1989; Watson et al., 1997); “B” in Figure 8b represents a basic amino acid in this motif, and X a hydropathic amino acid. Heparan sulfate proteoglycans, which are structurally related to heparin (Kim et al., 2017) and which are covering the extracellular surface of neurons, have been shown to play a crucial role in the unconventional export of tau (Katsinelos et al., 2018). We were therefore interested to test if Ng and its C‐terminal fragments carrying the RKKIK sequence bind to heparin beads, which could offer an explanation why only full‐length Ng and fragments C‐terminal to the Ng42/43 calpain cleavage site were so far found in CSF. Indeed, as shown in Figure 8a and b, only full‐length Ng and the C‐terminal fragments Ng43‐78 and Ng24‐48 (which all carry the RKKIK sequence) bound to heparin beads, but not the N‐terminal half of Ng (Ng1‐42). One caveat of this heparin bead binding experiment is that it cannot be ruled out that binding may have occurred to the agarose backbone itself (non‐coupled agarose beads of the same batch were not available for control). These initial binding results on beads need to be therefore confirmed by alternative methods (e.g., surface plasmon resonance). While it remains to be shown that the heparin‐binding motif of Ng mediates binding of Ng to heparan sulfate proteoglycans on the outer neuronal surface, it seems plausible that Ng and its C‐terminal fragments may share the same unconventional export mechanism with tau, which potentially could explain the strong correlation of CSF Ng with tau concentrations (Kvartsberg, Duits, et al., 2015). In opposition to this hypothesis, we have now also found N‐terminal Ng fragments which do not carry the presumed heparin‐binding motif. Either that motif is not relevant for the export of Ng species from the cell after all, or separate ways exist for N‐terminal fragments of Ng to appear in CSF.

### 4.1 Strengths and limitations

The analysis of different forms of Ng in CSF presented challenges from various perspectives: first, some forms are present in CSF only in very low quantities, some forms are difficult to detect by MS (larger MW), some bind only weakly to western blot membranes (small MW), and the choice of antibodies available for the N‐terminal region is very limited. However, a strength of this analysis is the combined use of those orthogonal techniques, western blot and IP‐MS to overcome the above‐mentioned sensitivity limitations of either technique. Because of the use of concentrated CSF and a modified western blot procedure, it was for the first time possible to directly detect neurogranin in CSF on immunoblots, without prior enrichment by IP. In the assay to estimate C‐terminal peptides by depleting CSF samples of N‐terminal and full‐length forms, we could directly target remaining C‐terminal Ng fragments without simultaneously measuring full‐length Ng. A limitation is that this assay gives an overall result comparing all C‐terminal Ng peptides to the “total‐Ng” (sum of full‐length and C‐terminal peptides) and is also not indicative of the amount of N‐terminal fragments that may be present as well. Another limitation is certainly the small sample size of only 20 samples, characterized by biochemical AD criteria only. Furthermore, there were no pre‐existing data on the ratio of C‐terminal fragments to total‐Ng in CSF from AD and control individuals on the basis of which a formal power calculation could have been done. Given the relatively high variability of Ng levels in the control and AD groups, our study should be seen as a pilot study, to be confirmed with higher sample numbers with both clinically and biochemically characterized AD and control samples, ideally by ELISA methods targeting directly only full‐length Ng and either N‐terminal or C‐terminal fragments along with full‐length Ng.

## 5 CONCLUSIONS

We detected different molecular forms of Ng present in CSF, which correspond to monomeric and both N‐and C‐truncated forms of Ng in CSF, as well as higher molecular weight forms of Ng, either representing Ng oligomers or Ng complexed with other proteins present in CSF. C‐terminal Ng peptides that are detectable by an in‐house ELISA but that do not bind to NG‐H6 constituted on average about 50% of all Ng in the 20 CSF samples studied, and no group differences in the ratio of C‐terminal fragments to total‐Ng among control and AD samples could be detected. Both full‐length Ng as well as C‐terminal fragments which carry the RKKIK sequence likely bind to heparin, which may allow their export from neurons via the unconventional mode of cellular export.

## 6 FUTURE PERSPECTIVES

The study highlights that several molecular forms of Ng are present in CSF; however, the determination of the composition of various higher molecular forms of Ng, for example, by mass spectrometry, is still hampered by the small amounts present in CSF and this is an area that still requires further efforts.

The quantitation of Ng and its fragments by ELISA has good potential to be utilized in biomarker assays to follow disease progression of AD. The current study indicated that the overall fragment/total‐Ng ratio did not differ between control and AD samples. Our analysis suggests that similar predictive power for Ng assays targeting different regions of Ng can be expected, even though absolute values for Ng may differ depending on the assay chosen as those assays target different Ng species. Similar predictive power in such assays has indeed been reported (Willemse et al., 2018), but this should be further confirmed on much larger sample sets. Moreover, it would be of great interest for the interpretation of pathogenic events or for the development of more specific biomarker assays to compare the peptide profiles of Ng in CSF (as determined by IP‐MS) among the various neurodegenerative diseases.

## 7 CONTRIBUTIONS

FHN and BB designed the study. FHN and BB planned and performed experiments including western blot, heparin binding, silver and Coomassie staining, size exclusion chromatography and ultrafiltration, analyzed, and interpreted data. FHN wrote the manuscript with guidance from BB. Brain samples were provided by TL and CET. EC prepared brain extracts and performed IP‐MS together with GB. All authors were involved in project discussions, provided intellectual input throughout the work and participated in manuscript revisions.

## CONFLICTS OF INTEREST

HZ has served at advisory boards of Denali, Roche Diagnostics, Samumed, CogRx and Wave, has given lectures in symposia sponsored by Fujirebio, Alzecure and Biogen, and is a co‐founder of Brain Biomarker Solutions in Gothenburg AB, a GU Ventures‐based platform company at the University of Gothenburg. KB has served as a consultant or at advisory boards for Alzheon, Abcam, Axon, Biogen, Eli Lilly, Merck, Novartis, Pfizer, and Roche Diagnostics, and is a co‐founder of Brain Biomarker Solutions in Gothenburg AB, a GU Ventures‐based platform company at the University of Gothenburg. The other authors report no conflicts of interest.

## Supporting information

Fig S1‐S4
