# Supplementary material for: Molecular forms of neurogranin in cerebrospinal fluid
Source: J Neurochem. 2020 Dec 17;157(3):816–33. doi: 10.1111/jnc.15252 (PMC8378242; doi:10.1111/jnc.15252)
Supplement: Supplementary file 1 — Fig S1‐S4 [file JNC-157-816-s001.pdf]

Molecular forms of neurogranin in cerebrospinal fluid

Supplementary Files

Faisal Hayat Nazir[1,*], Elena Camporesi[1,5], Gunnar Brinkmalm[1,5], Tammaryn Lashley[2,3], Christina E. Toomey[2,3,4], Hlin Kvartsberg[1,5], Henrik Zetterberg[1,3,4,5], Kaj Blennow[1,5], Bruno Becker[1,5,*]

[1]Institute of Neuroscience and Physiology, Department of Psychiatry and Neurochemistry, The Sahlgrenska Academy at the University of Gothenburg, Göteborg, Sweden
[2]Queen Square Brain Bank for Neurological Disorders, Department of Clinical and Movement Neuroscience, UCL Institute of Neurology, London, UK
[3]Department of Neurodegenerative Disease, UCL Institute of Neurology, Queen Square, London, UK
[4]UK Dementia Research Institute at UCL, London, WC1N 3BG, UK
[5]Clinical Neurochemistry Laboratory, Sahlgrenska University Hospital, Mölndal, Sweden
* Corresponding authors: Bruno Becker and Faisal Hayat Nazir

Supplementary Figure 1

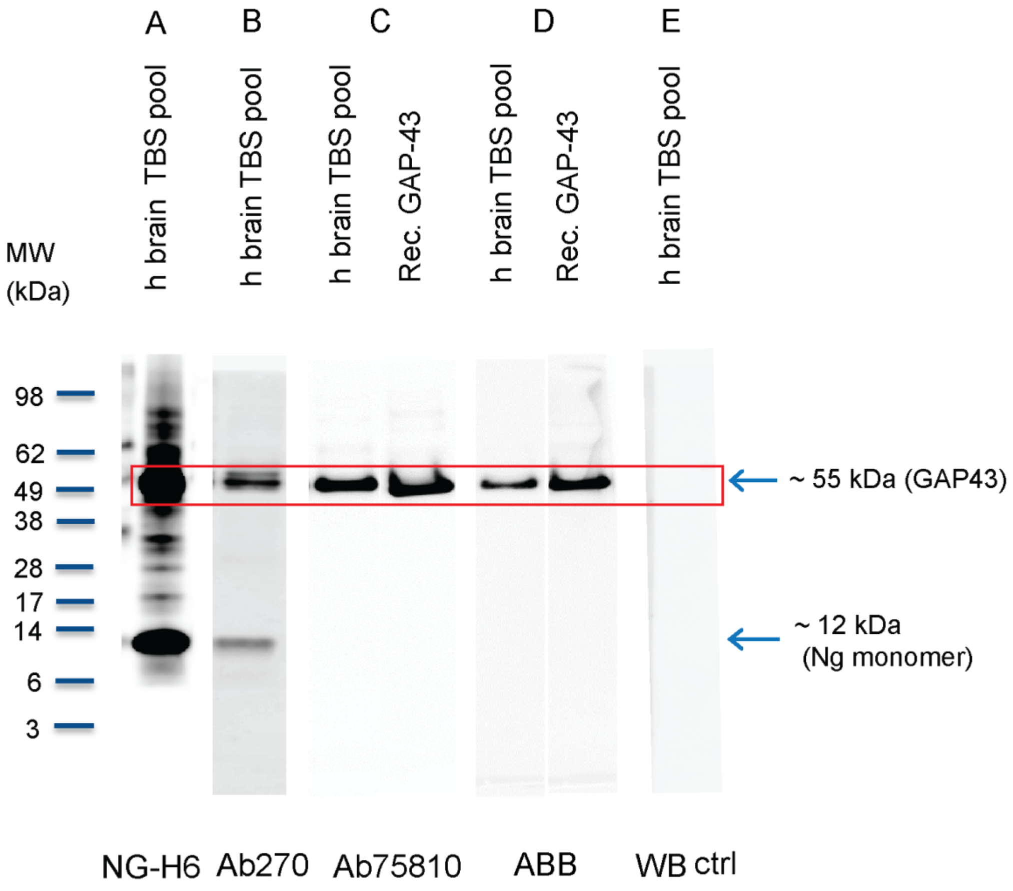

Supplementary Figure 1 The panels represent immunoblots of pooled TBS extracts of human brain and recombinant GAP-43. Samples were analyzed on 4-12% SDS-PAGE gels at non-reducing conditions. NG-H6, Ng mab **(panel A);** Ab270, Ng polyclonal antibody **(panel B)**; Ab75810, GAP-43 mab **(panel C);** ABB, GAP-43 polyclonal antibody **(panel D)**. Red rectangle denotes the GAP-43 bands detected and the arrows indicate the apparent molecular weights. The bands at 12 kDa in A and B represent Ng monomer. WB ctrl, western blot control, probed with rabbit IgG_K binding protein-HRP only **(panel E)**.

Supplementary Figure 2

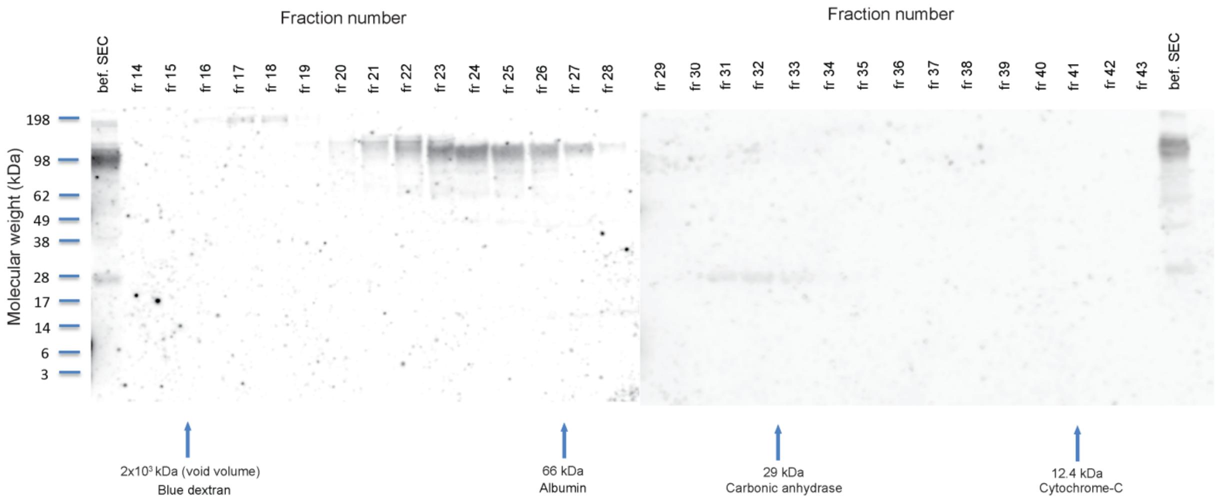

Supplementary Figure 2 Control blots to immunoblots shown in Figure 4. The control immunoblots shown were developed using anti-mouse IgG HRP-conjugated antibody only.

Supplementary Figure 3

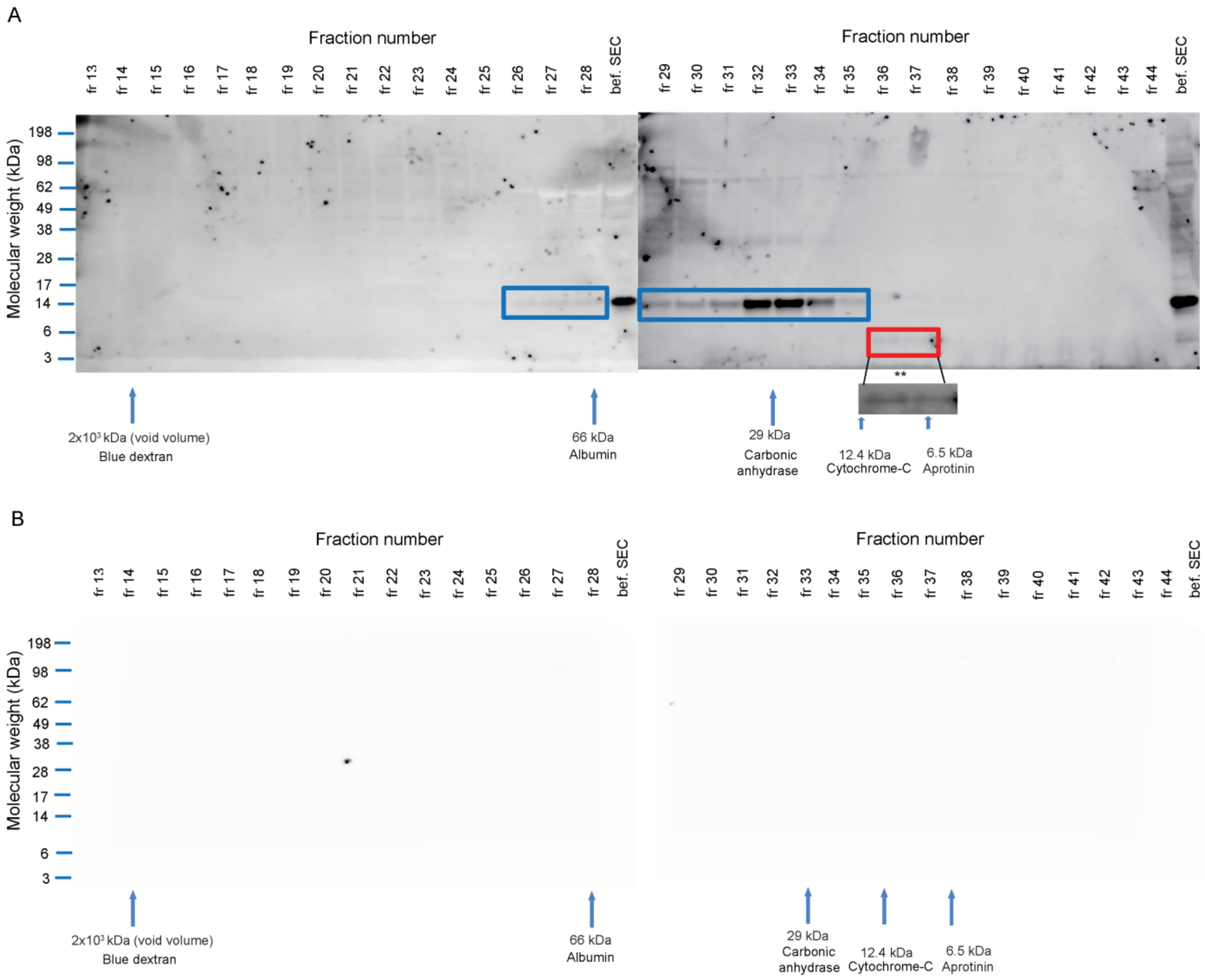

Supplementary Figure 3 Neurogranin is present in different molecular forms in CSF. (**A**) Western blots using NG36 as primary antibody. **(B)** Western blot control blots [as in **(A)**, but with NG36 omitted]. Size exclusion chromatography (SEC) fractions (#13-44) of a concentrated CSF pool were collected and processed for SDS-PAGE at reducing conditions followed by immunoblotting. Molecular weight markers are shown on the left side of each blot and the fraction numbers are shown above each lane on the blots. Fraction “before SEC” represents the concentrated CSF sample before SEC, but 2.5x less of this sample was applied as compared to the samples from the column fractions. The positions where SEC standards elute during calibration of the SEC column are indicated by arrows below the blots; blue dextran (marking void volume; 2,000 kDa), bovine serum albumin (66 kDa), carbonic anhydrase (from bovine erythrocytes; 29 kDa), cytochrome C (from horse heart; 12.4 kDa) and aprotinin (6.5 kDa). The rectangle shown in blue corresponds to ~12 kDa and the rectangle shown in red corresponds to ~6 kDa. The magnified part in **panel A** (**), shown at higher exposure, indicates Ng fragments corresponding to ~6 kDa.

Supplementary Figure 4

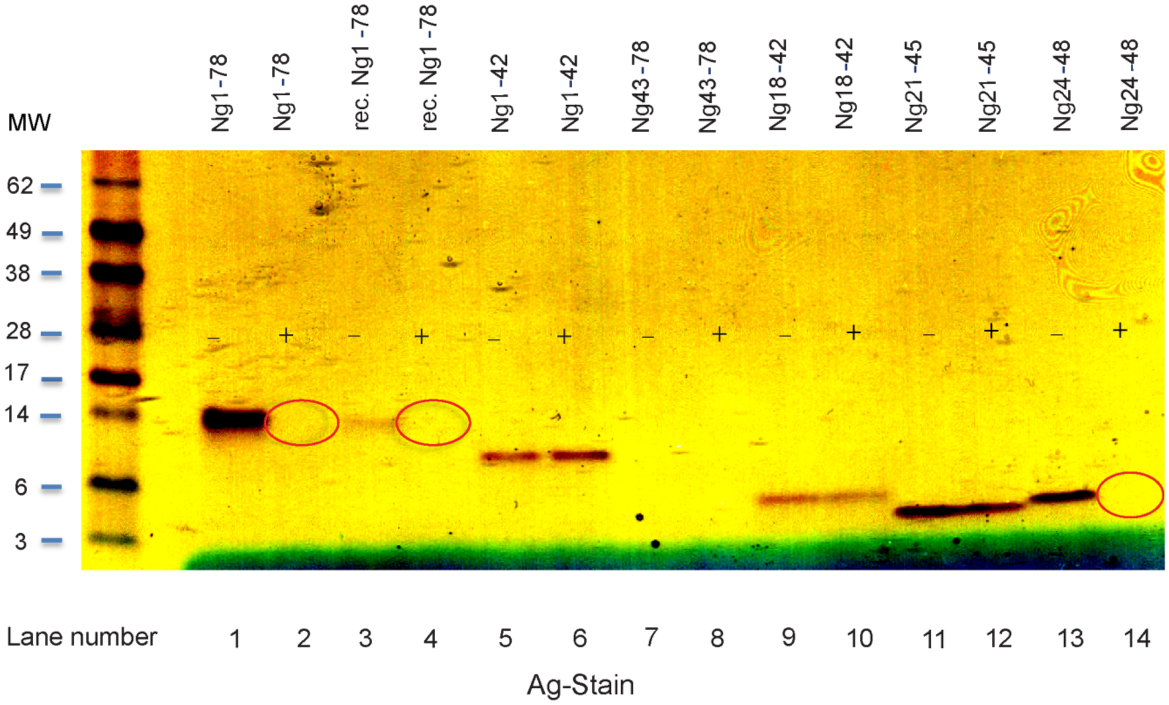

Supplementary Figure 4 Full-length Ng and C-terminal Ng peptides containing the KKIK aa sequence motif bind to heparin beads, but not to N-terminal peptides NgX...42. Synthetic and recombinant (rec.) Ng protein and peptides, either exposed to PBS control ("-" in gel image) or heparin gel ("+"), were run under reducing conditions on a 4-12% SDS PAGE gel. The gel was then stained with silver stain. The red circles point to the disappearance of the peptide bands due to binding to the heparin beads. MW, molecular weight markers (kDa). With Ag stain alone, it was not possible to determine binding of Ng43-78 to heparin because this positively charged peptide does not bind to silver ions, resulting in lack of detection. Therefore, the Ag stained gel was further stained with Coomassie dye to reveal also Ng43-78 (this final result is shown in Figure 8A in the main text).
